# Blood-based tumour mutation index act as prognostic predictor for immunotherapy and chemotherapy in non-small cell lung cancer patients

**DOI:** 10.1186/s40364-022-00400-5

**Published:** 2022-07-29

**Authors:** Jun Lu, Jun Wu, Yuqing Lou, Qin Shi, Jun Xu, Lele Zhang, Wei Nie, Jie Qian, Yanan Wang, Yanwei Zhang, Jing Jiao, Xueyan Zhang, Wei Zhang, Huimin Wang, Tianqing Chu, Hua Zhong, Baohui Han

**Affiliations:** 1 Department of Pulmonary Medicine, Shanghai Chest Hospital, Shanghai Jiao Tong University School of Medicine, Shanghai, 200030 China; 2 School of Life Science, East China Normal University, Shanghai, China; 3 Department of Oncology, Baoshan Branch of Shuguang Hospital, Shanghai University of Traditional Chinese Medicine, Shanghai, China; 4 Department of Emergency Medicine, The First Hospital of Anhui Medical University, Hefei, Anhui China; 5 Department of Emergency Medicine, Shanghai Chest Hospital, Shanghai Jiao Tong University School of Medicine, Shanghai, China; 6 Department of Imaging and Nuclear Medicine, Shanghai Chest Hospital, Shanghai Jiao Tong University School of Medicine, Shanghai, China

**Keywords:** Precision therapy, Circulating tumour DNA, Tumour mutation index, Non-small cell lung cancer, Liquid biopsy

## Abstract

**Background:**

Circulating tumour DNA (ctDNA)-based sequencing might provide a simple test for the stratified model of non-small cell lung cancer (NSCLC). Here, we aimed to assess the ctDNA sequencing-based tumour mutation index (TMI) model for screening responders (from non-responders) among NSCLC patients who received monotherapy with docetaxel or atezolizumab.

**Methods:**

We performed a retrospective analysis of the POPLAR (NCT01903993) and OAK (NCT02008227) trials. We identified three biomarkers, blood tumour mutation burden (bTMB), sensitive blood tumour mutation burden (sbTMB) and unfavourable mutation score (UMS), of the ctDNA profiles. After integrating the advantages and disadvantages of the three independent biomarkers, we developed the TMI model and identified NSCLC patients who may benefit from monotherapy with docetaxel or atezolizumab in terms of overall survival (OS).

**Results:**

The TMI model as a stratified biomarker for docetaxel responders provided a median OS duration of 5.55 months longer than non-responders in the OAK cohort, with a significantly decreased hazard ratio (HR). Moreover, atezolizumab responders had a 10.21-month OS advantage over atezolizumab non-responders in the OAK cohort via TMI stratification, and the HR was also decreased significantly. The TMI demonstrated effectiveness for stratifying responders in the POPLAR cohort. Importantly, we found that the TMI model could screen additional responders upon combining the cohorts from the POPLAR and OAK trials after adjustment.

**Conclusion:**

In the present study, we provide a novel TMI model for screening responders (from non-responders) among NSCLC patients who received the 2nd-line monotherapy with docetaxel or atezolizumab. We believe that the biomarker TMI will potentially be effective for the clinical treatment of NSCLC in the future.

**Supplementary Information:**

The online version contains supplementary material available at 10.1186/s40364-022-00400-5.

## Background

Clinical biomarkers for non-small cell lung cancer (NSCLC) can be used to address drug-related stratification and have contributed greatly to improving overall survival (OS) benefits [1–6]. With the continuous discovery of anti-cancer drugs for NSCLC, biomarkers that can be used for guiding clinical practice deserve further exploration [7, 8]. Chemotherapy plays an important role in the history of NSCLC treatment, and the viewpoint that it is suitable for all NSCLC patients currently holds true [8–10]. However, the development of next-generation sequencing (NGS) changed our viewpoint [11–14]. We previously found that patients harbouring a low blood tumour mutation burden (bTMB) received more OS benefits from docetaxel therapy than those harbouring a high bTMB [15]. In addition, for NSCLC patients who received immunotherapy and those with high levels of the biomarker-TMB harboured more neoantigens than those with low levels. Generally, high neoantigen levels contribute to inhibit the PD-L1/PD1 signalling pathway via immune checkpoint inhibitors, activate T cells and kill cancer cells [16, 17]. Therefore, two biomarkers (TMB and PD-L1) have been proposed for immunotherapy stratification in the clinic [18–20]. However, according to later retrospective noncomprehensive screening studies of these two biomarkers [21], more optimized biomarkers should be developed for clinical practice.

The potential causes for the dissatisfaction for the present biomarkers might be attributed to various aspects of cancer patients. The biological characteristics of NSCLC cells determine the complexity of their genetic variation and tumour-associated microenvironment (TAM) [17, 22]. This complexity has been demonstrated in clinical practice and helps explain why NSCLC patients with the same pathological type receive different OS benefits from the same therapeutic regimen. With the development of precision medicine, NSCLC patients harbouring driver gene mutations (such as EGFR/ROS1/ALK/KRAS) have received corresponding tyrosine kinase inhibitor (TKI) therapy [23, 24]. However, patients who do not harbour the above driver gene mutations receive other therapeutic regimens, such as chemotherapy, immunotherapy, and anti-angiogenic therapy [3, 6, 10, 25]. Therefore, the exploration of biomarkers for these therapeutic regimens is an important means to promote precision therapy for NSCLC in clinical practice.

Circulating tumour DNA (ctDNA)-based sequencing provides a potential platform for these therapeutic regimens, and its validity has been demonstrated in the past 5 years [3, 11, 13, 14, 26, 27]. Nonetheless, the biomarker derived from ctDNA profiling for tumour treatment is also closely related to tumour heterogeneity and temporal tumour evolution [14]. Therefore, the ideal biomarker not only embodies the factors of genetic variation, the TAM, heterogeneity and temporal tumour evolution but also includes the patient’s clinical characteristics, such as sex, smoking history, driver gene status, pathological type, and number of metastases. Based on this hypothesis, we integrated clinical characteristics and genomic profiles, developed a predictive model of the tumour mutation index (TMI), and described effective clinical stratification in patients with advanced NSCLC who received anti-angiogenic therapy [3]. However, whether the biomarker TMI (or optimized TMI) could be used to guide chemotherapy and immunotherapy remained unclear. Therefore, in the present study, we mainly explored the underlying stratification validity of the TMI for guiding chemotherapy and immunotherapy in NSCLC patients.

## Methods

### Patients

In this study, all the sequencing data and clinical data were obtained from the POPLAR (NCT01903993) and OAK (NCT02008227) trials [10, 26, 28]. The PD-L1 status was not analysed in the initial study of all enrolled NSCLC patients from the OAK trial or POPLAR trial (*n =* 1137). However, the PD-L1 status was analysed in the retrospective study, but only in patients from the OAK trial. In the POPLAR cohort, 143 patients received docetaxel therapy, and 144 received atezolizumab therapy. In the OAK cohort (850 patients), half of the patients received docetaxel therapy, and the other half received atezolizumab therapy. According to the sequencing data, we first filtered the patients who did not pass quality control (QC), and the biomarker-evaluable population (BEP) did not achieve a minimum of 800× sequence coverage. Finally, 211 NSCLC patients from the POPLAR trial (106 who received docetaxel and 105 who received atezolizumab) and 642 NSCLC patients from the OAK trial (318 who received docetaxel and 324 who received atezolizumab) remained for the subsequent analysis.

### Calculation of blood tumour mutation burden (bTMB) and sensitive blood tumour mutation burden (sbTMB)

The mutation information on circulating tumour DNA (ctDNA) for all blood samples was obtained in accordance with a previous study [26]. The effects of individual somatic mutations were defined as missense, synonymous, splice and nonsense. Here, we counted the number of mutations that were classified as missense, synonymous, splice and nonsense for each patient, which was defined as bTMB. Moreover, we calculated the mutations that were classified as missense and splice, and defined as sbTMB.

### Calculation of the unfavourable mutation score (UMS)

In addition to bTMB and sbTMB, we also introduced the UMS as another biomarker, which was calculated as follows. We first performed survival analysis for each mutated gene and found that 44 genes were associated with OS benefits (*P*-value: 0–0.3) in the patients in the OAK cohort who received docetaxel. According to the different correlations, we divided these 44 genes into 5 levels and assigned the scores according to the *P*-value as follows: a *P*-value between 0 and 0.05 was scored as 5; a *P*-value between 0.05 and 0.1 was scored as 4; a *P*-value between 0.1 and 0.15 was scored as 3; a *P*-value between 0.15 and 0.2 was scored as 2; and a *P*-value between 0.2 and 0.3 was scored as 1. All of these genes were defined as unfavourable mutated genes. If one patient harboured multiple unfavourable mutated genes, the UMS was calculated according to their *P*-value. Finally, survival analysis was performed using the UMS in patients from the OAK cohort. Similarly, we performed survival analysis for each mutation and found that 40 genes were associated with OS benefits in the patients in the OAK cohort who received atezolizumab (*P*-value: 0–0.3). The UMS of each patient who received atezolizumab was calculated as described above.

### Cut-off analysis

Following our previous study [3], we determined the optimal cut-off for each biomarker (bTMB, sbTMB and UMS) using Log-rank analysis. Briefly, for each biomarker, we performed survival analysis with different cut-offs, and the optimal cut-off was regarded as the cut-off that led to the lowest *P*-value. This strategy was applied to determine both progression-free survival (PFS) and OS.

#### bTMB, sbTMB, and UMS for clinical stratification

The procedures used for cut-off determination were performed as described above. Kaplan-Meier curves were generated to evaluate the correlation between mutational burden and response to docetaxel. The lowest *P*-value was selected as the cut-off *P*-value. Determination of the ongoing response and survival status was described as “clinical efficacy analysis”. The significance of the *P-*value was calculated by comparing the median PFS or median OS durations between those with high levels of biomarkers (bTMB, sbTMB, and UMS) and those with low levels of biomarkers. The receiver operating characteristic (ROC) curves for predicting PFS and OS were generated by the cut-off *P-*values of these biomarkers using GraphPad Prism. The area under the curve (AUC) (95% CI) and null hypothesis test *P*-value were determined by ROC curve analysis. For the atezolizumab cohort, two cut-off values were selected for the biomarker bTMB and the biomarker sbTMB. Kaplan-Meier curves were generated to evaluate the correlation between mutational burden and response to atezolizumab, and then the lowest *P*-value was set as the first cut-off *P*-value. Serial curves were generated to analyse the correlation between the cut-off value and PFS/OS, and the value after intersection was set as the second cut-off *P*-value. The other analyses of clinical stratification were performed similarly to those in the docetaxel cohort.

#### TMI generation

We first divided the patients in the discovery cohort (OAK cohort) into two groups (high risk and low risk) according to the different biomarkers (bTMB, sbTMB and UMS) along with their clinical characteristics, including sex (male or female), smoking history (smokers or non-smokers), pathological subtype (lung squamous cell carcinoma [LUSC] or non-LUSC), driver gene mutation (positive or negative) and number of metastases (≥3 or none). For each clinical characteristic, we calculated the optimal thresholds along with the hazard ratio (HR) for bTMB, sbTMB, and UMS, respectively. For each patient, we calculated the score of HR for each clinical characteristic (male or female, smokers or non-smokers, LUSC or non-LUSC, driver gene positive or negative, number of metastases ≥3 or none, respectively) under different individual biomarker of bTMB, sbTMB, and UMS, and then the sum of the score of HRs was defined as the TMI. The median TMI in the discovery cohort was used as the final threshold to divide the patients into low TMI and high TMI groups.

#### Response analysis and HRs in subgroups using the biomarkers bTMB, sbTMB, and UMS

In the discovery cohort (OAK cohort), we performed the analyses of response to docetaxel and atezolizumab on different subgroups (male, female, non-smoker, smoker, non-LUSC, LUSC, driver gene (+), driver gene (−), < 3 metastases, and ≥ 3 metastases) using the biomarkers bTMB, sbTMB, and UMS. Then, we performed Kaplan-Meier curve analysis of PFS/OS, calculated the log-rank *P-*value and HR for each biomarker, performed ROC curve analysis, and calculated the AUC and null hypothesis test *P-*values.

#### Discovery and validation analyses of the TMI

According to the criteria described above, a TMI score was obtained for each patient based on their sequencing data and clinical characteristics. Then, the stratification value (the cut-off was set as the median value) and the HR were calculated based on the patient’s TMI score. Finally, we compared the differences in TMI stratification between different subgroups and analysed its potential stratified value and defects.

#### Statistical analysis

The log-rank test was used to compare Kaplan-Meier curves during TMI generation and in the validation cohort and subsequently in the stratification analysis. The HRs and exact 95% CIs are reported. An unpaired t test was used to compare the mutational burden between “responders” and “non-responders”. The ROC curve was determined by plotting the rate of responders at various cut-off settings of predictors. That is, the proportion of all responders with a mutational burden above any given cut-off point (sensitivity) was plotted against the proportion of non-responders who would also exceed the same cut-off point (1 – specificity). The AUCs and exact 95% CIs are reported. To examine the credibility of the stratification, a null hypothesis test was performed to analyse the ROC curve. Statistical analyses were performed using GraphPad Prism 5. Differences were considered significant at *P < 0.05, **P < 0.01, and ***P < 0.001.

## Results

**Fig. 1:**
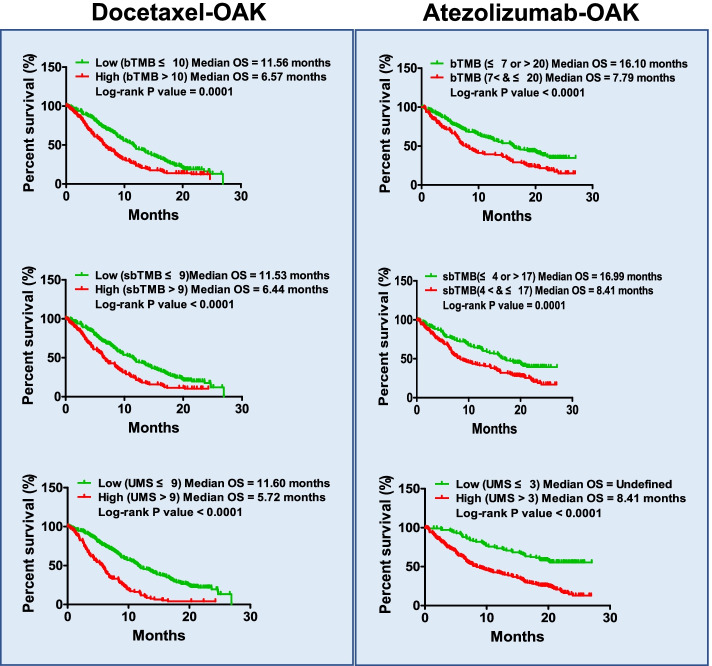
TMB and UMS were used as biomarkers for docetaxel and atezolizumab stratification in the discovery cohort. Left Kaplan-Meier plots of OS in NSCLC patients receiving docetaxel when using the biomarkers bTMB, sbTMB and UMS. The optimal cut-off values of the three biomarkers were determined for stratification. OS curves of responders and non-responders are shown on the top left (bTMB, responders: *n =* 165; non-responders: *n =* 153), middle left (sbTMB, responders: *n =* 181; non-responders: *n =* 137), and bottom left (UMS, responders: *n =* 207; non-responders: *n =* 111). Right Kaplan-Meier plots of OS in NSCLC patients receiving atezolizumab when using the biomarkers bTMB, sbTMB and UMS. The cut-off values of the three biomarkers were set as follows: bTMB: ≤ 7 or > 20; sbTMB: ≤ 4 or > 17; and UMS ≤ 3. OS curves of responders and non-responders are shown on the top right (bTMB, responders: *n =* 129; non-responders: *n =* 195), middle right (sbTMB, responders: *n =* 147; non-responders: *n =* 177), and bottom right (UMS, responders: *n =* 102; non-responders: *n =* 222)

The POPLAR trial and OAK trial provided excellent ctDNA sequencing data and clinical data. First, we performed a correlation analysis of PFS and bTMB in patients from the POPLAR cohort who received docetaxel, and the results indicated that patients with bTMB ≤5 received more PFS benefit than patients with bTMB > 5 (median PFS: 6.11 months vs 2.83 months, *P =* 0.0041) (Supplementary Fig. 1). Furthermore, we performed OS analysis and obtained a similar result but with a higher predictive value (AUC: 0.71 vs 0.65) (Supplementary Fig. 2). While we performed a similar analysis on docetaxel-treated patients from the OAK cohort, the optimal cut-off value of bTMB was different. Patients with bTMB ≤10 received a greater benefit than patients with bTMB > 10 (median PFS: 4.40 months vs 2.79 months, *P* < 0.0001; median OS: 11.56 months vs 6.57 months, *P =* 0.0001) (Fig. 1, top left; Supplementary Fig. 3). The subgroup analysis based on the clinical characteristics suggested that the biomarker bTMB could not be used to stratify the docetaxel responders in some subgroups (PFS analysis: female and driver gene (+); OS analysis: non-smoker, driver gene (+), and ≥ 3 metastases) (Supplementary Table 1). These results suggest that bTMB could be used as a biomarker to screen docetaxel responders, but its efficacy for subgroup stratification should be further improved.

To explore alternative biomarkers, we removed synonymous and nonsense mutations and calculated the missense and splice mutation burdens (i.e., sbTMB). OS analysis in the POPLAR cohort suggested that patients with sbTMB ≤4 received a greater benefit (median OS: 14.39 months vs 7.75 months, *P* < 0.0001), while in the OAK cohort, patients with sbTMB ≤9 received a greater benefit (median OS: 11.53 months vs 6.44 months, *P* < 0.0001) (Fig. 1, middle left; Supplementary Fig. 4). Furthermore, our results showed that sbTMB can be used to screen docetaxel responders effectively in subgroups from the OAK cohort during PFS and OS analyses (Supplementary Table 1). These results indicate that sbTMB could be used as a biomarker to stratify docetaxel responders from non-responders. Nevertheless, based on the results presented above, it is interesting that the optimal cut-off value of not only the biomarker bTMB but also of the biomarker sbTMB, was different between the POPLAR cohort and the OAK cohort (Supplementary Figs. 1–4). These results indicate that more optimized biomarkers should be screened to stratify responders in both the POPLAR cohort and the OAK cohort.

Each patient in the OAK cohort harboured at least 1 somatic mutation. Do these mutations result in different benefits from docetaxel therapy? To answer this question, we performed a correlation analysis between each mutated gene and OS and found that 44 genes were associated with the benefit of docetaxel therapy (correlation *P-*value: 0.0–0.3) (Supplementary Fig. 5A). By calculating the UMS and performing OS analysis for each patient, we found that patients with a low UMS based on ctDNA sequencing results received a greater benefit from docetaxel monotherapy than those with a high UMS (Supplementary Fig. 5B-E). Here, we selected the “optimal cut-off value = 9” and performed OS analysis on the OAK cohort. The results suggested that patients with a UMS ≤ 9 received a greater benefit from docetaxel therapy than patients with a UMS > 9 (median OS: 11.60 months vs 5.72 months, *P* < 0.0001) (Fig. 1, bottom left). PFS and OS analyses of responders suggested that the UMS could be used to screen docetaxel responders effectively for nearly all subgroups except for the non-smoker subgroup (Supplementary Table 1). These results suggest that the UMS potentially be used as a biomarker for docetaxel stratification. Overall, the above three biomarkers (bTMB, sbTMB, and UMS) showed different advantages in different subgroups, suggesting that their advantages could be integrated into one model for predicting docetaxel responders.

On the other hand, to understand the underlying stratified value of the above biomarkers (bTMB, sbTMB, and UMS) in immunotherapy, we performed a similar analysis on patients in the POPLAR cohort and the OAK cohort who received atezolizumab monotherapy. Different from docetaxel monotherapy, patients in the POPLAR cohort with bTMB > 18 received a greater PFS benefit from atezolizumab therapy (PFS: low = 2.14 months vs high = 4.98 months, *P =* 0.0103), but patients with bTMB ≤3 received a greater OS benefit from atezolizumab therapy (median OS: low = 21.52 months vs high = 10.09 months, *P =* 0.0190) (Supplementary Fig. 6 and 7). The curve of low bTMB and the curve of high bTMB intersect with each other in either the PFS analysis or the OS analysis for cut-off determination. This phenomenon indicated that the patients harbouring a lower mutational burden or a higher mutational burden received a greater benefit from atezolizumab therapy than the patients harbouring a middle mutational burden (Supplementary Fig. 6A and 7A).

Furthermore, we performed a similar analysis on the OAK cohort and found that patients with bTMB ≤7 or > 20 received a greater benefit from atezolizumab therapy than patients with 7 > bTMB ≤20 (median PFS: 3.91 months vs 1.54 months, *P* < 0.0001; median OS: 16.10 months vs 7.79 months, *P* < 0.0001) (Fig. 1, top right; Supplementary Fig. 8). Kaplan-Meier curve analysis of PFS indicated that this biomarker could not achieve significant stratification on the following subgroups: female, non-smoker, and driver gene (+). For the OS analysis, these results changed to the following subgroups: non-smoker, LUSC, and driver gene (+) (Supplementary Table 2). These results suggest that more complexity exists in blood ctDNA sequencing-guided atezolizumab stratification than in guided docetaxel stratification.

To understand the potential predictive value of the biomarker sbTMB in patients who received atezolizumab therapy, we also performed Kaplan-Meier curve analysis on patients in the POPLAR cohort and the OAK cohort. The results suggested that patients in the POPLAR cohort with bTMB ≤4 or > 12 received a greater OS benefit, while patients in the OAK cohort with bTMB ≤4 or > 17 received a greater OS benefit (median OS: 16.99 months vs 8.41 months, *P* < 0.0001) (Fig. 1, middle right; Supplementary Fig. 9). Subgroup analysis in the OAK cohort showed that except for the three subgroups females, driver gene (+) and ≥ 3 metastases, the other subgroups could be stratified effectively via sbTMB for PFS. However, these subgroups (non-smoker, LUSC, and driver gene (+)) could not be stratified effectively via the biomarker sbTMB during OS analysis. These results suggest that sbTMB can also be used as a potential biomarker for screening atezolizumab responders.

In the OAK cohort, similar to the docetaxel cohort, we found that 40 gene mutations were associated with OS outcomes (correlation *P-*value: 0.0–0.3) in the atezolizumab cohort (Supplementary Fig. 10A). Our analysis suggested that patients with a low UMS received more OS benefits than those with a high UMS. After optimizing the cut-off settings, we found that patients with a low UMS (UMS ≤ 3) received a significant OS benefit (median OS: undefined vs 8.41 months, *P* < 0.0001) (Fig. 1, bottom right; Supplementary Fig. 10B-E). For the OS subgroup analysis, nearly all subgroups except for the driver gene (+) could be stratified via the biomarker UMS. However, only four subgroups (smoker, LUSC, driver gene (−) and < 3 metastases) could be stratified by the use of the UMS in the PFS analysis (Supplementary Table 2). Therefore, we hypothesize that a predictive model can be constructed for the clinical stratification of docetaxel or atezolizumab responders through integrating both the advantages and disadvantages of the above biomarkers (bTMB, sbTMB, and UMS).

**Fig. 2:**
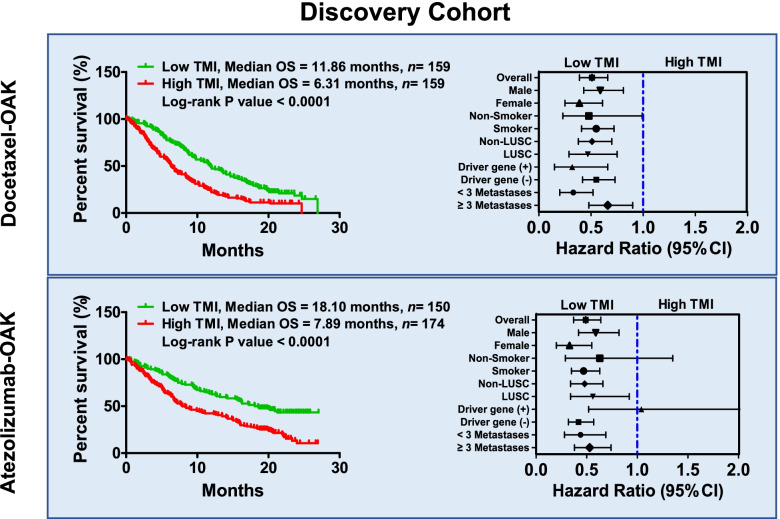
The TMI was used for docetaxel and atezolizumab stratification in the discovery cohort (OAK cohort). Top Kaplan-Meier plots of OS in NSCLC patients receiving docetaxel when the TMI cut-off was set at the median value. For responders, the median OS duration was 11.86 months (*n =* 159); for non-responders, the median OS duration was 6.31 months (*n =* 159). The HRs of all patients and the corresponding subgroups are shown on the top right. Bottom Kaplan-Meier plots of OS in NSCLC patients receiving atezolizumab when the TMI cut-off was set at the median value. For responders, the median OS duration was 18.10 months (*n =* 150); for non-responders, the median OS duration was 7.89 months (*n =* 174). The HRs of all patients and the corresponding subgroups are shown on the lower right

To validate this hypothesis, we developed a TMI model that integrates the advantages and disadvantages of the above three biomarkers (Supplementary Fig. 11). After calculation, each patient was assigned a TMI score. When the cut-off was set at the median value, docetaxel monotherapy-treated patients in the OAK cohort with a low TMI received a greater OS benefit than those with a high TMI (median OS: 11.86 months vs 6.31 months, *P* < 0.0001). After stratification, we found that the HR of patients with a low TMI decreased significantly (HR = 0.51, 95% CI 0.39–0.66) compared to that of patients with a high TMI. This phenomenon was demonstrated in both all patients and in the patient subgroups (Fig. 2, top). For patients who received atezolizumab therapy, when the cut-off was set at the median value, those with a low TMI received a greater OS benefit than those with a high TMI (median OS: 18.10 months vs 7.89 months, *P* < 0.0001). Similar to the docetaxel cohort, the HR of patients with a low TMI was markedly decreased compared with that of patients with a high TMI (HR = 0.49, 95% CI 0.37–0.64). These results suggest that the TMI may be an ideal biomarker for predicting both docetaxel and atezolizumab responders among NSCLC patients.

**Fig. 3:**
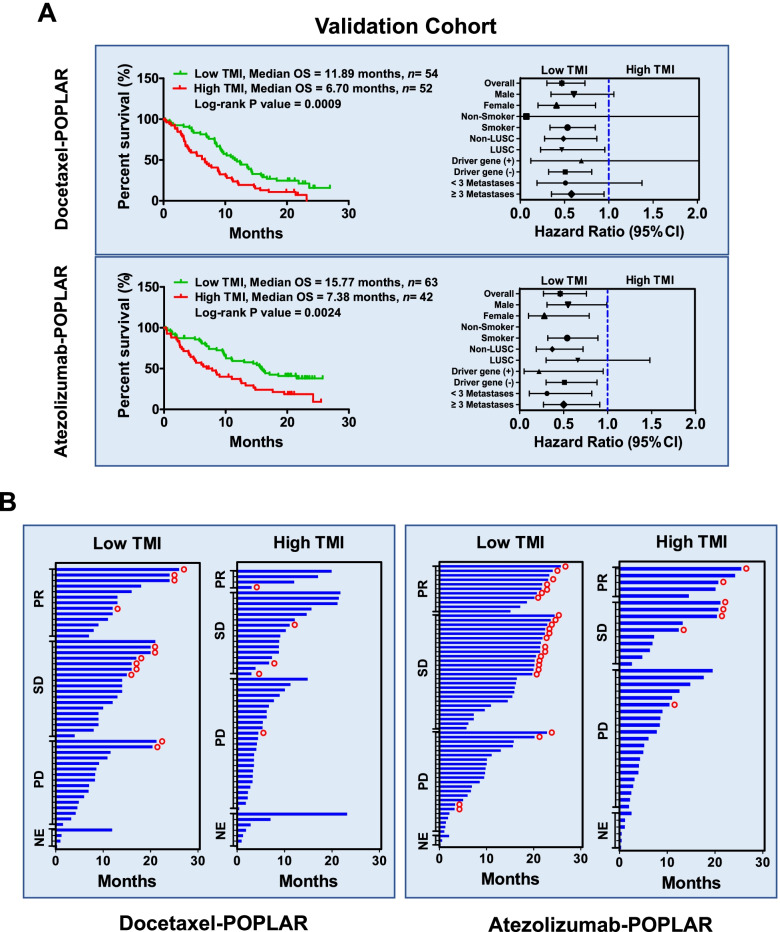
Validation of the effectiveness of the TMI in the validation cohort (POPLAR cohort). A Top Kaplan-Meier plots of OS in NSCLC patients receiving docetaxel after TMI stratification. For responders, the median OS duration was 11.89 months (*n =* 54); for non-responders, the median OS duration was 6.70 months (*n =* 52). The HRs of all patients and the corresponding subgroups are shown on the top right. Bottom Kaplan-Meier plots of OS in NSCLC patients receiving atezolizumab after TMI stratification. For responders, the median OS duration was 15.77 months (*n* = 63); for non-responders, the median OS duration was 7.38 months (*n* = 42). The HRs of all patients and the corresponding subgroups are shown on the lower right. B Left Evaluation of clinical efficacy in patients defined as having a low TMI and a high TMI after receiving docetaxel monotherapy. Right Evaluation of clinical efficacy in patients defined as having a low TMI and a high TMI after receiving atezolizumab monotherapy. The blue pillar represents the survival time for each patient; the red circle represents those still alive at the end of the follow-up

To test whether the biomarker TMI can be used to screen responders in an independent cohort, we assigned the POPLAR cohort as the validation cohort. The validated results revealed that patients with a low TMI received more OS benefit either from docetaxel monotherapy or atezolizumab monotherapy (docetaxel, median OS: low TMI = 11.89 months vs high TMI = 6.70 months, *P =* 0.0009; atezolizumab, median OS: low TMI = 15.77 months vs high TMI = 7.38 months, *P =* 0.0024). This results also indicated that TMI has comprehensive advantages during predicting responders (from non-responders) who received monotherapy with docetaxel or atezolizumab, especially with comprehensive advantage in predicting atezolizumab-responders while compared with those individual biomarkers (bTMB, sbTMB, UMS) (Fig. 3A, Supplementary Fig. 12, Supplementary Table 3 and 4). This phenomenon was also validated when HR distribution was examined. (Fig. 3A, Supplementary Fig. 13 and 14). Patients with a low TMI received more efficacy evaluations of partial response (PR) rate, stable disease (SD), and survival time (Fig. 3B). These results suggested TMI model could be used to stratify responders to both the docetaxel therapeutic regimen and the atezolizumab therapeutic regimen.

**Fig. 4:**
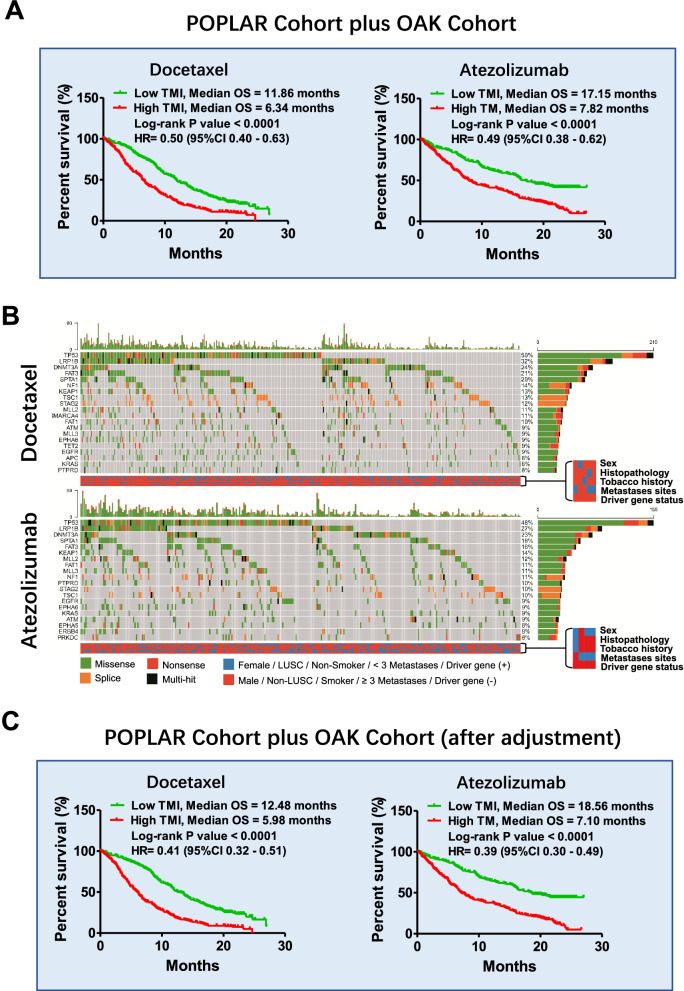
Adjusted TMI for screening responders in the combined POPLAR and OAK cohorts. A TMI-based stratification of docetaxel-treated or atezolizumab-treated NSCLC patients from the POPLAR cohort plus the OAK cohort. B Mutation patterns and clinical characteristics of NSCLC patients who received docetaxel and atezolizumab. C Kaplan-Meier curves of the adjusted TMI-based stratification of NSCLC patients who received monotherapy with docetaxel or atezolizumab

The above results were further validated in all enrolled NSCLC patients (POPLAR cohort plus OAK cohort) (Fig. 4A, Supplementary Fig. 18). TMI-based stratification worked well in both the PD cohort and the SD cohort as well as in the TC0/1/2 and IC0/1/2 or TC3 and IC3 cohorts (Supplementary Fig. 15). Mutation patterns between the docetaxel cohort and the atezolizumab cohort were similar (including the top 10 or 20 mutated genes, mutated types, single nucleotide variant [SNV] class, etc.) (Fig. 4B, Supplementary Fig. 16). Furthermore, in the low TMI cohort, we found many patients with an OS duration < 6 months, while in the high TMI cohort, we found many patients with an OS duration > 18 months. By comparing genomic information, we found that specific mutations were associated with the above phenomenon under the TMI-stratified system (Supplementary Fig. 17). More importantly, either the NSCLC patients received docetaxel or received atezolizumab, the patients harbouring low TMI obtained the most OS benefit from atezolizumab therapy (Fig. 4, Supplementary Fig. 18).

## Discussion

Biomarkers have played an important role in the clinical treatment of NSCLC [1]. The discovery of biomarkers from venous blood is considered a simple and effective means to guide the use of anti-cancer agents [3, 11, 13, 14, 24, 26]. The blood-based TMI model that we developed previously demonstrated its efficacy in screening responders to the anti-angiogenic agent anlotinib [3], but whether the stratified model could be used to screen responders among NSCLC patients who received docetaxel or atezolizumab was unclear. Therefore, we sought to perform clinical stratification via the TMI in these patients, and our results suggested that the biomarker TMI is an effective stratification model to screen docetaxel responders and atezolizumab responders. This is the first demonstration that the TMI can be accurately and reproducibly calculated after plasma ctDNA sequencing and that the TMI is associated with the OS benefit of monotherapy with docetaxel or atezolizumab.

TMB calculated from tumour tissue sequencing or blood ctDNA sequencing is correlated with OS benefits in patients who receive chemotherapy or immunotherapy [15, 26]. However, further improvement is needed to calculate the mutational burden from tissue DNA or blood ctDNA for guiding chemotherapy or immunotherapy. The phenomenon of heterogeneity exists in nearly all types of tumours, and clones and subclones with somatic mutations differ according to their location in the tumour [22]. Heterogeneity may be associated with the response rate of anti-cancer agents [14, 22]. In addition, other clinical characteristics (including sex, smoking history, pathological type, driver gene status and number of metastases) may also be associated with the response rate to anti-cancer agents. Here, we hypothesized that a computational model that integrated the factors of sequencing data and clinical characteristics could be used to screen responders. In our previous study, we developed a TMI model and showed excellent stratification of advanced NSCLC patients who received anlotinib therapy [3]. However, as a multi-target TKI, anlotinib shows significant differences in its molecular anti-cancer mechanism compared to other anti-cancer agents, including the chemotherapy agent docetaxel and the immune checkpoint inhibitor atezolizumab. To understand whether the TMI model could also work well for these two agents, we performed similar analyses on the two famous clinical trials (POPLAR and OAK) in which the blood-based sequencing data and clinical data have been made public. In the present study, we first evaluated the stratification characteristics of three individual biomarkers, bTMB, sbTMB, and UMS, in patients who received docetaxel. Our results suggested that the above three biomarkers could be used to screen responders (including PFS and OS) from non-responders, either from the POPLAR cohort or the OAK cohort. Although the optimal cut-off value was different between the two cohorts, the overall stratification characteristics showed that the lower levels of bTMB, sbTMB and UMS were, the greater the OS benefit the patient received from docetaxel therapy. Subgroup analysis indicated that each biomarker has dissatisfaction and is different from other biomarkers. How to compensate for these deficiencies and highlight the advantages of these biomarkers are important issues. Therefore, we developed a TMI model that integrated the advantages of the three biomarkers to screen responders much more effectively. The results indicated that responders could be screened effectively by use of the biomarker-TMI in both the discovery cohort (OAK) and the validation cohort (POPLAR), and the HRs of responders were decreased significantly in both all patients and patient subgroups.

For patients who received the immune checkpoint inhibitor atezolizumab, the phenomenon was slightly different from that of patients who received monotherapy with docetaxel or anlotinib [3]. It is interesting that the in the POPLAR cohort, patients with bTMB > 18 obtained a greater PFS benefit while patients with bTMB ≤3 obtained a greater OS benefit after atezolizumab therapy. However, in the OAK cohort, we found that patients with a low and high bTMB (≤ 7 or > 20) obtained a greater benefit (including PFS and OS) from atezolizumab therapy. The characteristics of the biomarker-sbTMB are similar to those of the biomarker-bTMB. For UMS, the characteristics were similar to those of the docetaxel cohort. This phenomenon resulted in difficulty establishing a TMI model for predicting atezolizumab responders. Therefore, we developed an autoscreening programme according to the characteristics of atezolizumab and established a TMI model in the discovery cohort (OAK). Using the TMI as a stratification biomarker, responders could be screened effectively in both the discovery cohort (OAK) and the validation cohort (POPLAR), and the HRs of responders were decreased significantly in both all patients and patient subgroups.

To date, the TMI model or optimized TMI model has demonstrated its effectiveness for stratifying NSCLC patients who respond to the anti-angiogenic agent anlotinib, the chemotherapy agent docetaxel, and the immune checkpoint inhibitor atezolizumab. The simple computational model described herein potentially guide clinical practice in the future. Nevertheless, this is just the beginning. The advantages of TMI might be included as follows: 1. Multiple levels of genomic variations were considered for developing the TMI model. 2. Different clinical characteristics (sex, smoking history, driver gene status, pathological type, and number of metastases) were included in the TMI model establishment. 3. More patients can be screened out to suggest receiving chemotherapy or immunotherapy via the biomarker-TMI as compared to bTMB [15, 26]. However, this model should be enriched and validated on many more clinical samples and features, more flexible procedures, and more types of cancer. Therefore, we summarized that the shortcomings of TMI model are potentially included as follows: 1. An independent validation should be performed on an outer cohort to test the predictive value. 2. as *p*-value only reveals the statistical significance, whether mean that the lower it is, the stronger the association is should be further validated. 3. Whether the Cox proportional hazard model is more suitable for ROC analysis need to be further tested. Nevertheless, as NGS continues to be used to guide clinical practice, a better TMI model will be developed to screen responders to chemotherapy, immunotherapy or multi-target TKI therapy.

In summary, we developed a TMI model that could be used as a stratified biomarker for guiding docetaxel and atezolizumab therapy in NSCLC patients. Whether the TMI model can be used as a biomarker for guiding anti-cancer agents in other types of cancer deserves further testing and attention.

## Supplementary Information

**Additional file 1.**

## Data Availability

The data and material of OAK and POPLAR study were derived from a previous publication, which was publicly available at https://www.nature.com/articles/s41591-018-0134-3. The source code of TMI generation as well as the code used to generate data and figures are available at https://github.com/Junwu302/NSCLC_TMI.

## Abbreviations

ctDNA: Circulating tumour DNA
NSCLC: Non-small cell lung cancer
bTMB: Blood tumour mutation burden
sbTMB: Sensitive blood tumour mutation burden
UMS: Unfavourable mutation score
OS: Overall survival
HR: Hazard ratio
NGS: Next-generation sequencing
TAM: Tumour-associated microenvironment
TKI: Tyrosine kinase inhibitor
QC: Quality control
BEP: Biomarker-evaluable population
PFS: Progression-free survival
ROC: Receiver operating characteristic
AUC: Area under the curve
LUSC: Lung squamous cell carcinoma
SNV: Single nucleotide variant
