# Supplementary material for: Blood-based tumour mutation index act as prognostic predictor for immunotherapy and chemotherapy in non-small cell lung cancer patients
Source: Biomark Res. 2022 Jul 29;10:55. doi: 10.1186/s40364-022-00400-5 (PMC9336041; doi:10.1186/s40364-022-00400-5)
Supplement: Supplementary file 1 — Additional file 1. [file 40364_2022_400_MOESM1_ESM.pdf]

Supplementary materials

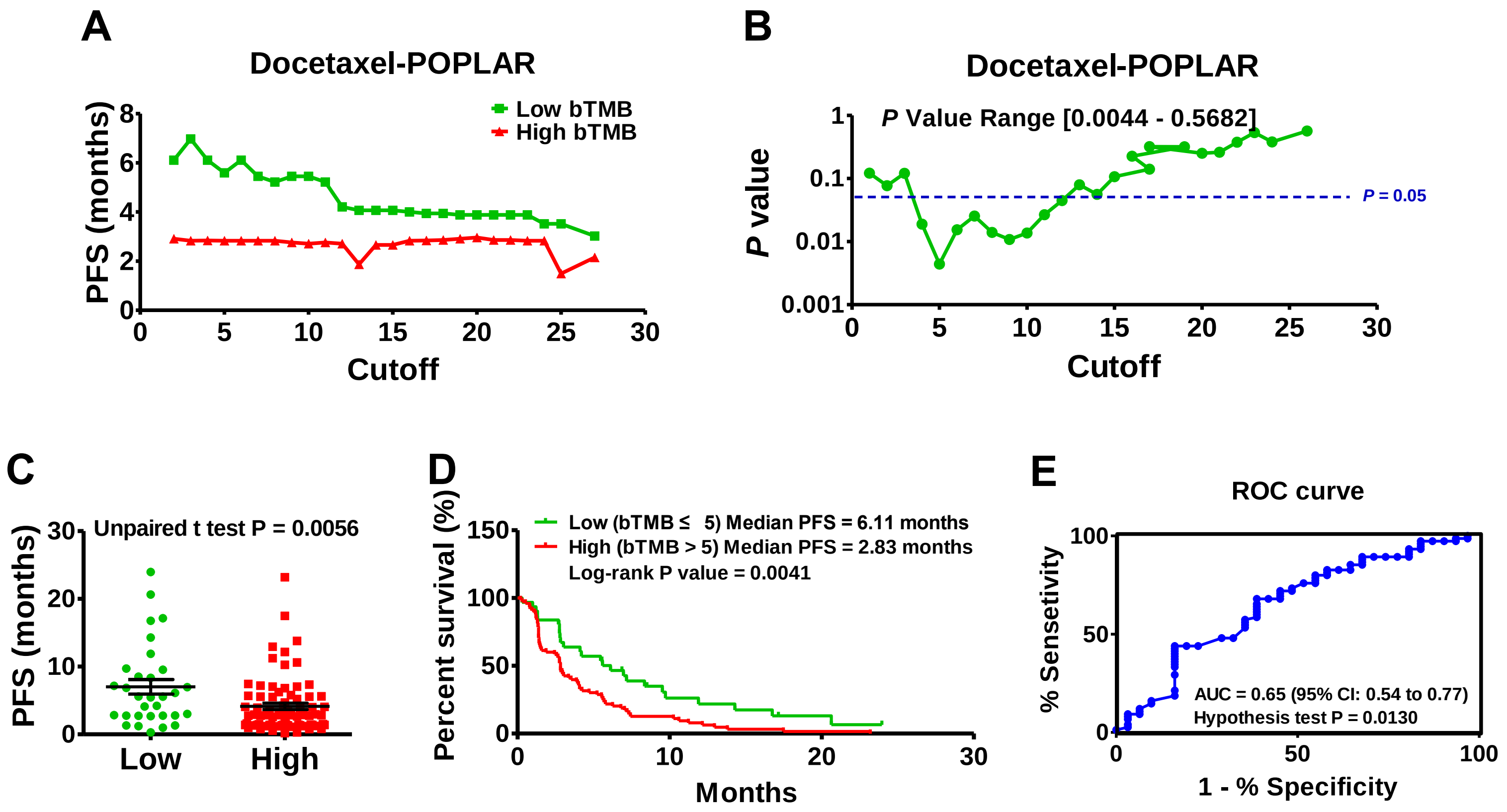

Supplementary Figure 1. Progression-free survival cut-off analysis of the biomarker bTMB for NSCLC patients in the POPLAR cohort who received docetaxel. (A) The correlation between bTMB and progression-free survival (PFS). Patients with a low bTMB had a better response to docetaxel therapy than those with a high bTMB. **(B)** The alteration in each *P*-value when the cut-off value was changed. The cut-off value ranged between 1 and 27. The optimal *P*-value was 0.0044 when the cut-off value was set at 5. **(C)** Absolute PFS analysis between patients with a low bTMB (*n*= 31) and high bTMB (*n*= 75) (PFS: unpaired t test *P*= 0.0056). **(D)** Kaplan-Meier plots of PFS in NSCLC patients receiving docetaxel when the bTMB cut-off was set at 5. Patients with a low bTMB (*n*= 31) compared to those with a high bTMB (*n*= 75) (PFS: 6.11 months vs 2.83 months, log-rank *P*= 0.0041). **(E)** ROC curve for the correlation of bTMB with response to docetaxel. The AUC of PFS response prediction was 0.65 (95% CI 0.54 to 0.77, null hypothesis test *P*= 0.0130).

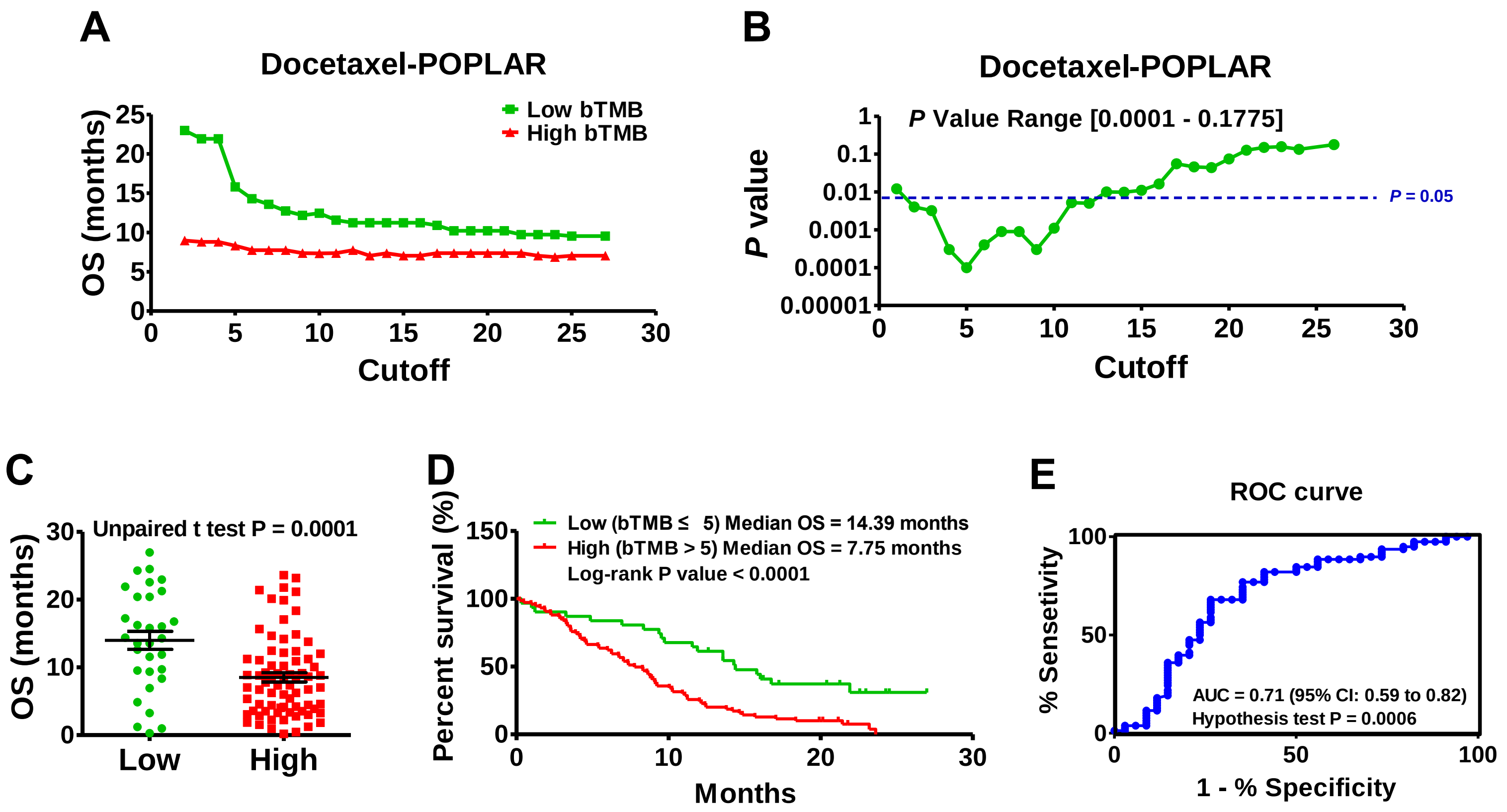

Supplementary Figure 2. Overall survival cut-off analysis of the biomarker bTMB for NSCLC patients in the POPLAR cohort who received docetaxel. (A) The correlation between bTMB and overall survival (OS). Patients with a low bTMB had a greater OS benefit after docetaxel therapy than those with a high bTMB. **(B)** The alteration in each *P*-value when the cut-off value was changed. The cut-off value ranged between 1 and 27. The optimal *P*-value was less than 0.0001 when the cut-off value was set at 5. **(C)** Absolute OS analysis between patients with a low bTMB (*n*= 31) and high bTMB (*n*= 75) (PFS: unpaired t test *P*= 0.0001). **(D)** Kaplan-Meier plots of OS in NSCLC patients receiving docetaxel when the bTMB cut-off was set at 5. Patients with a low bTMB (*n*= 31) compared to those with a high bTMB (*n*= 75) (OS: 14.39 months vs 7.75 months, log-rank *P* < 0.0001). **(E)** ROC curve for the correlation of bTMB with response to docetaxel. The AUC of OS response prediction was 0.71 (95% CI 0.59 to 0.82, null hypothesis test *P*= 0.0006).

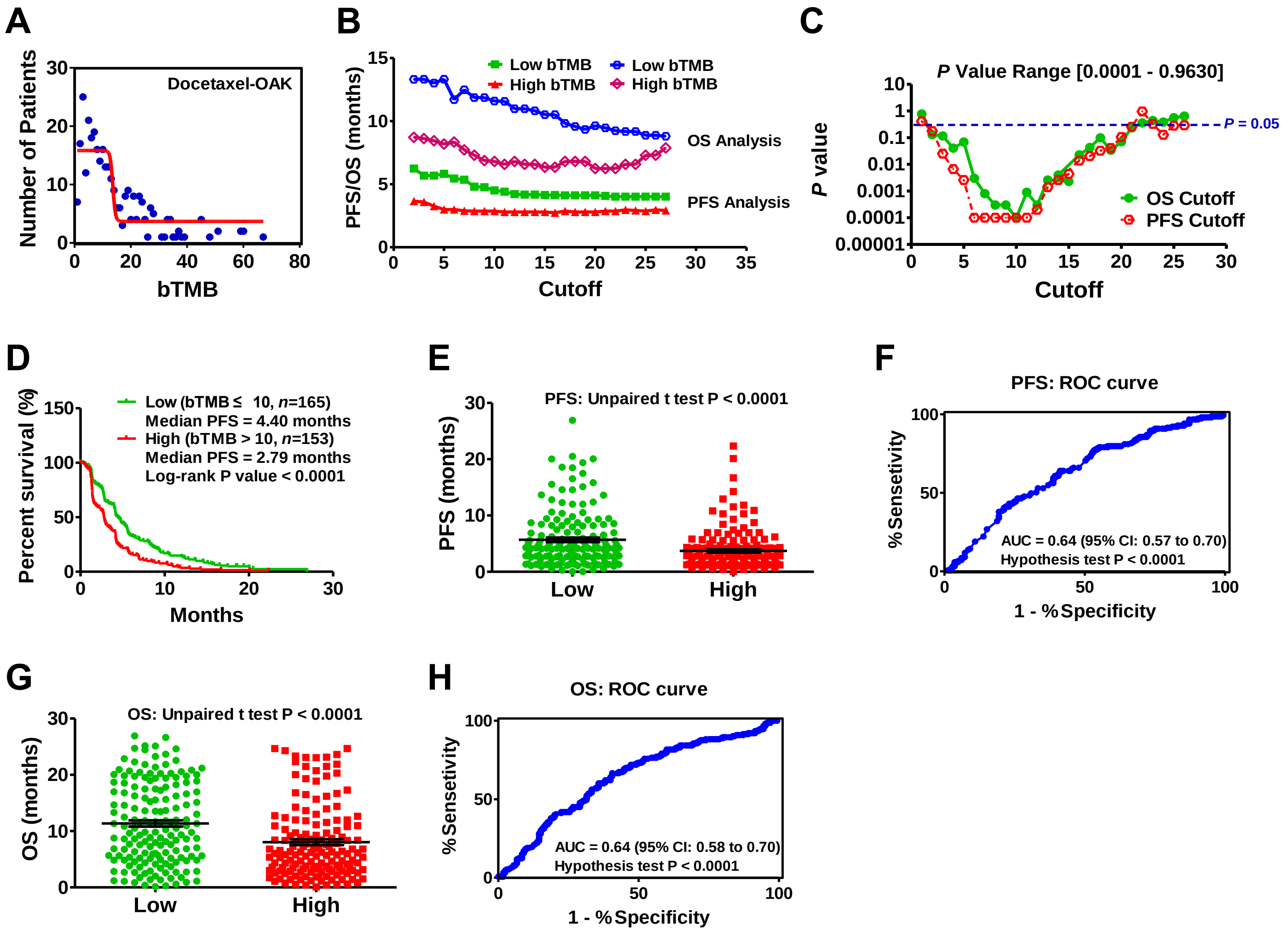

Supplementary Figure 3. Cut-off analysis of the biomarker bTMB for NSCLC patients in the OAK cohort who received docetaxel. (A) The distribution of bTMB. **(B)** The correlation between bTMB and survival time (PFS and OS). Patients with a low bTMB benefitted more (PFS and OS) from docetaxel therapy than those with a high bTMB. **(C)** The alteration in each *P*-value when the cut-off value was changed. The cut-off value ranged between 1 and 27. The optimal *P*-value was less than 0.0001 when the cut-off value was set at 10. **(D)** Kaplan-Meier plots of PFS in NSCLC patients receiving docetaxel when the bTMB cut-off was set at 10. Patients with a low bTMB (*n*= 165) compared to those with a high bTMB (*n*= 153) (OS: 4.40 months vs 2.79 months, log-rank *P* < 0.0001). **(E)** Absolute PFS analysis between patients with a low

bTMB (*n*= 165) and high bTMB (*n*= 153) (PFS: unpaired t test $P < 0.0001$). **(F)** ROC curve for the correlation of bTMB with response to docetaxel. The AUC of PFS response prediction was 0.64 (95% CI 0.57 to 0.70, null hypothesis test $P < 0.0001$). **(G)** Absolute OS analysis between patients with a low bTMB (*n*= 165) and high bTMB (*n*= 153) (OS: unpaired t test $P < 0.0001$). **(H)** ROC curve for the correlation of bTMB with response to docetaxel. The AUC of OS response prediction was 0.64 (95% CI 0.58 to 0.70, null hypothesis test $P < 0.0001$).

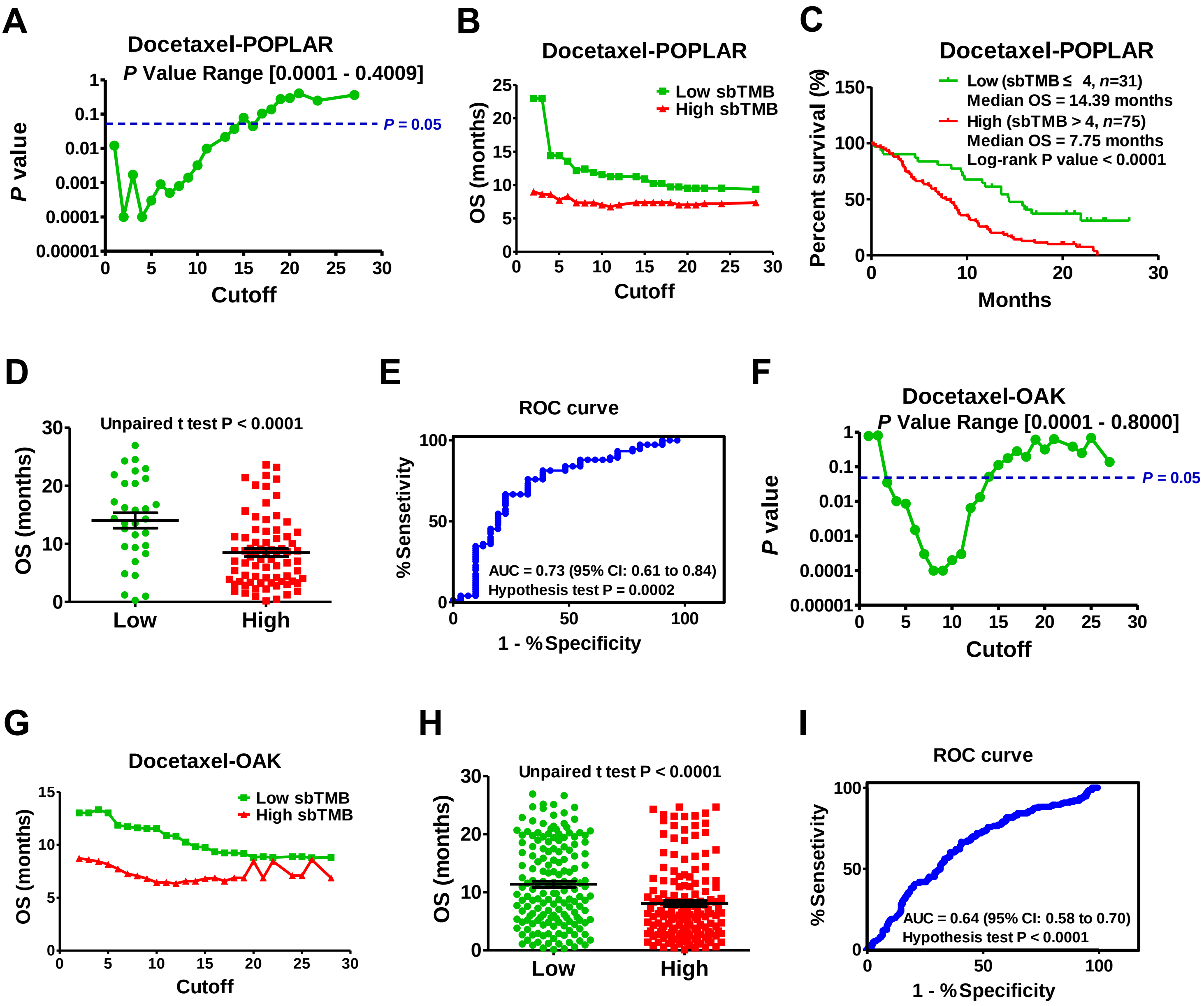

Supplementary Figure 4. Cut-off analysis of the biomarker sbTMB for NSCLC patients in the POPLAR and OAK cohorts who received docetaxel. (A) The alteration in each *P*-value when the cut-off value was changed in the POPLAR cohort. The cut-off value ranged between 1 and 27. The optimal *P*-value was less than 0.0001 when the cut-off value was set at 4. **(B)** The correlation between sbTMB and OS in the POPLAR cohort. Patients with a low sbTMB had a greater OS benefit after docetaxel therapy than those with a high sbTMB. **(C)** Kaplan-Meier plots of OS in NSCLC patients in the POPLAR cohort receiving docetaxel when the sbTMB cut-off was set at

4. Patients with a low sbTMB (*n*= 31) compared to those with a high sbTMB (*n*= 75) (OS: 14.39 months vs 7.75 months, log-rank $P < 0.0001$). **(D)** Absolute OS analysis between patients in the POPLAR cohort with a low sbTMB (*n*= 31) and high sbTMB (*n*= 75) (OS: unpaired t test $P < 0.0001$). **(E)** ROC curve for the correlation of sbTMB with response to docetaxel in the POPLAR cohort. The AUC of OS response prediction was 0.73 (95% CI 0.61 to 0.84, null hypothesis test $P= 0.0002$). **(F)** The alteration in each *P*-value when the cut-off value was changed in the OAK cohort. The cut-off value ranged between 1 and 27. The optimal *P*-value was less than 0.0001 when the cut-off value was set at 9. **(G)** The correlation between sbTMB and OS in the OAK cohort. Patients with a low sbTMB had a greater OS benefit after docetaxel therapy than those with a high sbTMB. **(H)** Absolute OS analysis between patients with a low bTMB (*n*= 165) and high bTMB (*n*= 153) (OS: unpaired t test $P < 0.0001$). **(I)** ROC curve for the correlation of bTMB with response to docetaxel. The AUC of OS response prediction was 0.64 (95% CI 0.58 to 0.70, null hypothesis test $P < 0.0001$).

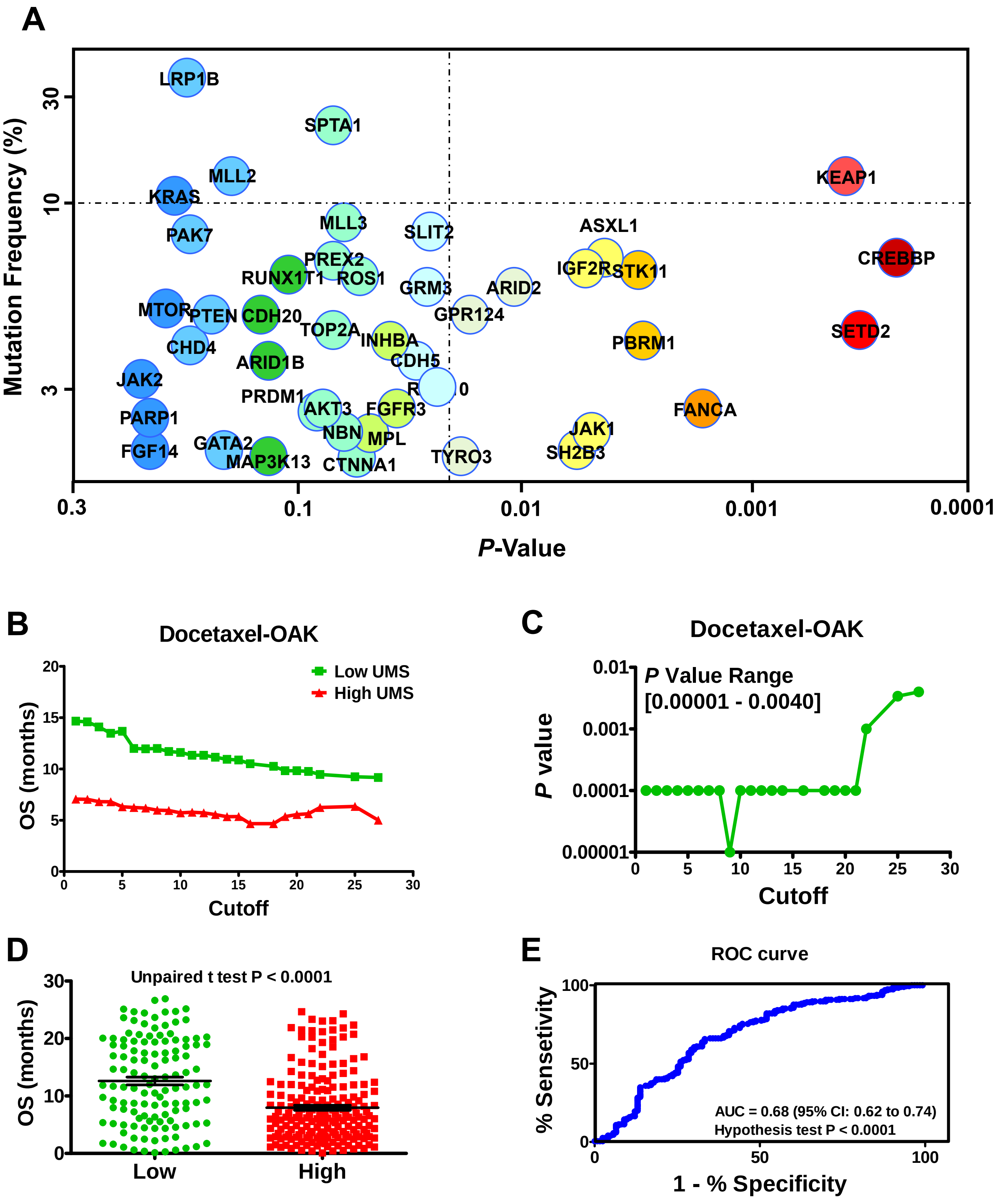

Supplementary Figure 5. Cut-off analysis of the biomarker UMS for NSCLC patients in the OAK cohort who received docetaxel. (A) The correlation analysis

between mutated genes and OS. Mutated frequency and significant *P*-value were showed. **(B)** The correlation between the UMS and OS. Patients with a low UMS had a greater OS benefit after docetaxel therapy than those with a high UMS. **(C)** The alteration in each *P*-value when the cut-off value was changed in the OAK cohort. The cut-off value ranged between 1 and 27. The optimal *P*-value was less than 0.00001 when the cut-off value was set at 9. **(D)** Absolute OS analysis between patients in the OAK cohort with a low UMS (n= 123) and a high UMS (n= 195) (OS: unpaired t test $P < 0.0001$). **(E)** ROC curve for the correlation of the UMS with response to docetaxel. The AUC of OS response prediction was 0.68 (95% CI 0.62 to 0.74, null hypothesis test $P < 0.0001$).

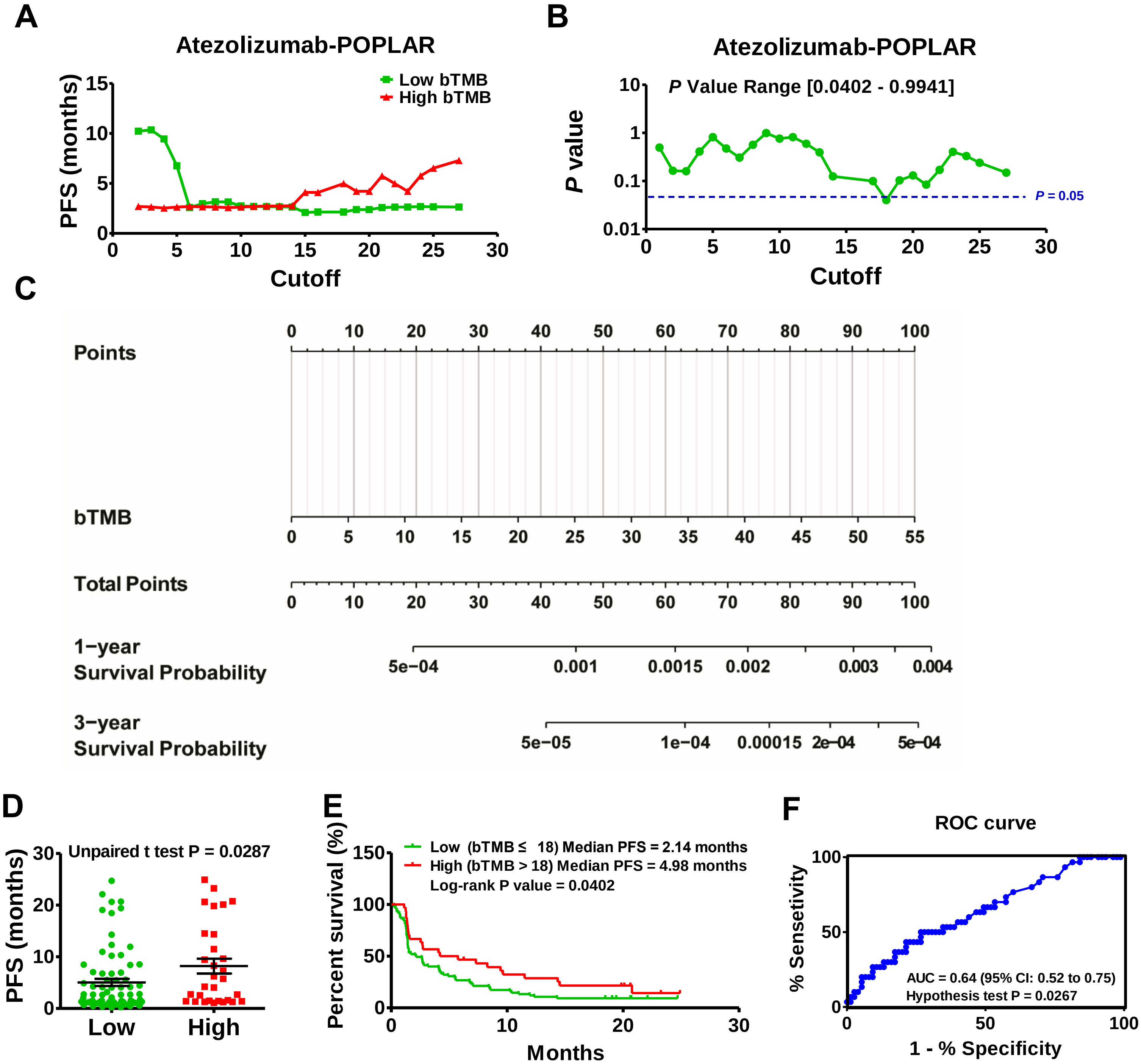

Supplementary Figure 6. PFS cut-off analysis of the biomarker bTMB for NSCLC patients in the POPLAR cohort who received atezolizumab. (A) The correlation between bTMB and PFS. Patients with a low bTMB or high bTMB had a better response to atezolizumab therapy than those with a median bTMB. **(B)** The alteration in each *P*-value when the cut-off value was changed. The cut-off value ranged between

1 and 27. The optimal *P*-value was 0.0402 when the cut-off value was set at 18. **(C)** The nomogram showed the correlation between bTMB and PFS. **(D)** Absolute PFS analysis between patients with a low bTMB (*n*= 75) and high bTMB (*n*= 30) (PFS: unpaired t test *P*= 0.0287). **(E)** Kaplan-Meier plots of PFS in NSCLC patients receiving atezolizumab when the bTMB cut-off was set at 18. Patients with a low bTMB (*n*= 75) compared to those with a high bTMB (*n*= 30) (PFS: 2.14 months vs 4.98 months, log-rank *P*= 0.0402). **(F)** ROC curve for the correlation of bTMB with response to atezolizumab. The AUC of PFS response prediction was 0.64 (95% CI 0.52 to 0.75, null hypothesis test *P*= 0.0267).

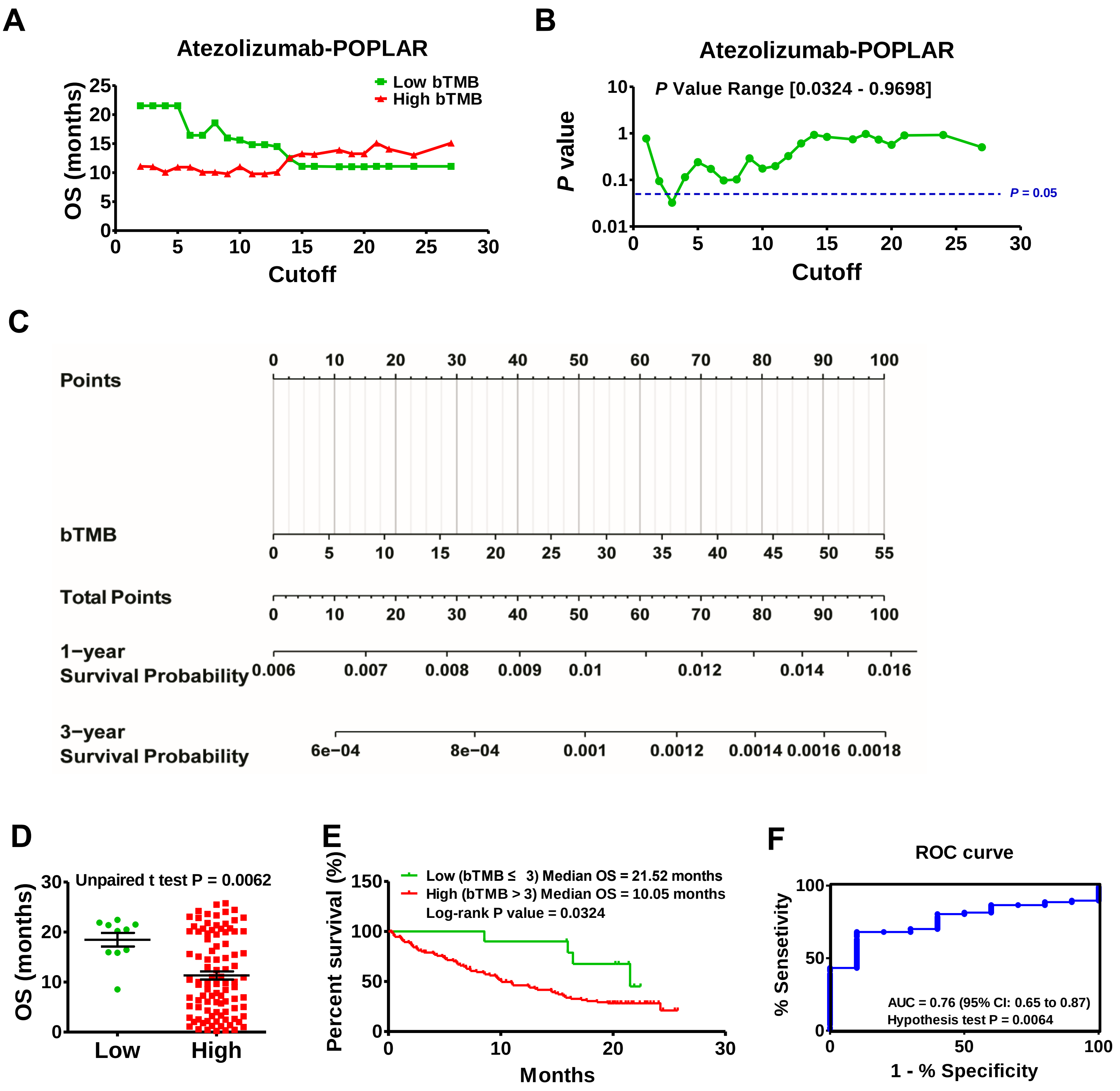

Supplementary Figure 7. OS cut-off analysis of the biomarker bTMB for NSCLC patients in the POPLAR cohort who received atezolizumab. (A) The correlation between bTMB and OS. Patients with a low bTMB or high bTMB had a greater OS

benefit after atezolizumab therapy than those with a moderate bTMB. **(B)** The alteration in each *P*-value when the cut-off value was changed. The cut-off value ranged between 1 and 27. The optimal *P*-value was less than 0.0324 when the cut-off value was set at 3. **(C)** The nomogram showed the correlation between bTMB and OS. **(D)** Absolute OS analysis between patients with a low bTMB (*n*= 10) and high bTMB (n= 95) (PFS: unpaired t test *P*= 0.0001). **(E)** Kaplan-Meier plots of OS in NSCLC patients receiving atezolizumab when the bTMB cut-off was set at 3. Patients with a low bTMB (*n*= 10) compared to those with a high bTMB (*n*= 95) (OS: 21.52 months vs 10.05 months, log-rank *P*= 0.0324). **(F)** ROC curve for the correlation of bTMB with response to atezolizumab. The AUC of OS response prediction was 0.76 (95% CI 0.65 to 0.87, null hypothesis test *P*= 0.0064).

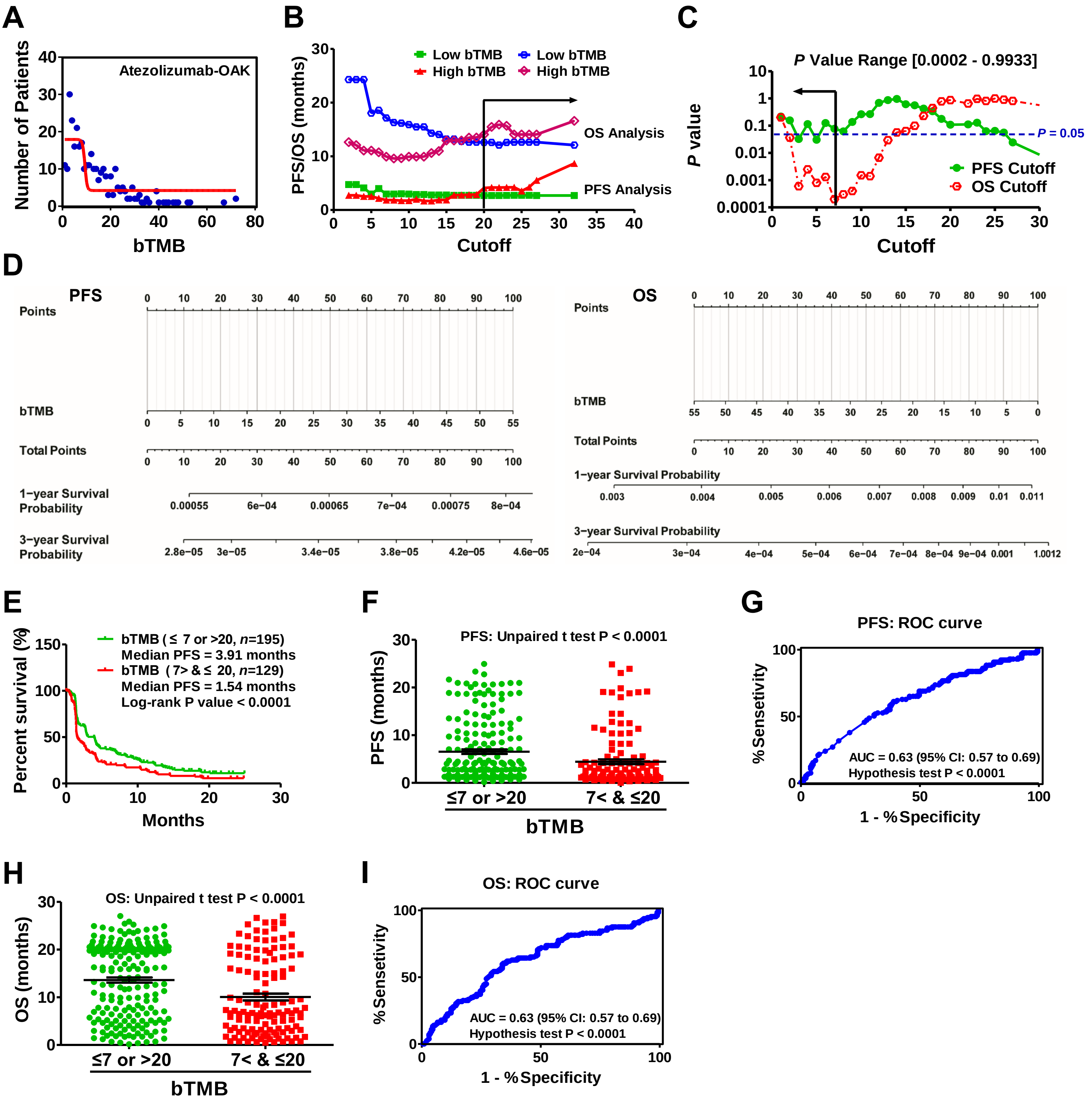

Supplementary Figure 8. Cut-off analysis of the biomarker bTMB for NSCLC patients in the OAK cohort who received atezolizumab. (A) The distribution of bTMB. **(B)** The correlation between bTMB and survival time (PFS and OS). Patients with a low bTMB or high bTMB (bTMB > 20) had greater PFS and OS benefits after

atezolizumab monotherapy than those with a moderate bTMB. **(C)** The alteration in each *P*-value when the cut-off value was changed. The cut-off value ranged between 1 and 27. The optimal *P*-value was 0.0002 when the cut-off value was set at 7. **(D) Left:** The nomogram showed the correlation between bTMB and PFS. **Right:** The nomogram showed the correlation between bTMB and OS. **(E)** Kaplan-Meier plots of PFS in NSCLC patients receiving atezolizumab when the bTMB cut-off was set at bTMB ≤7 or >20. Patients with bTMB ≤7 or >20 (*n*= 195) compared to those with 7< bTMB ≤20 (*n*= 129) (OS: 3.91 months vs 1.54 months, log-rank *P* < 0.0001). **(F)** Absolute PFS analysis between patients with bTMB ≤7 or >20 (*n*= 195) and those with 7< bTMB ≤20 (*n*= 129) (PFS: unpaired t test *P* < 0.0001). **(G)** ROC curve for the correlation of bTMB with response to atezolizumab. The AUC of PFS response prediction was 0.63 (95% CI 0.57 to 0.69, null hypothesis test *P* < 0.0001). **(H)** Absolute OS analysis between patients with bTMB ≤7 or >20 (*n*= 195) and those with 7< bTMB ≤20 (*n*= 129) (OS: unpaired t test *P* < 0.0001). **(I)** ROC curve for the correlation of bTMB with response to atezolizumab. The AUC of OS response prediction was 0.63 (95% CI 0.57 to 0.69, null hypothesis test *P* < 0.0001).

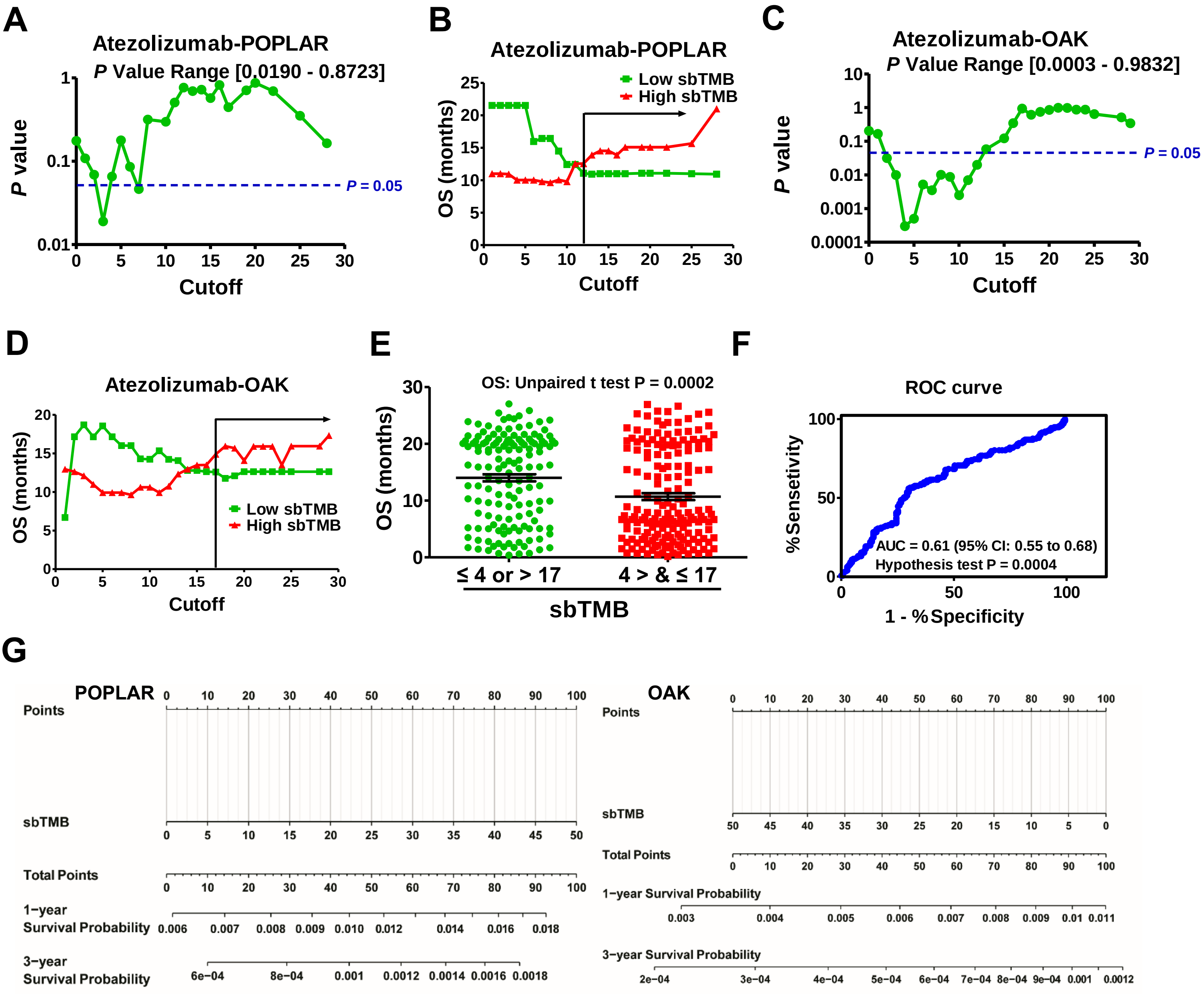

Supplementary Figure 9. Cut-off analysis of the biomarker sbTMB for NSCLC patients in the POPLAR and OAK cohorts who received atezolizumab. (A) The alteration in each *P*-value when the cut-off value was changed in the POPLAR cohort. The cut-off value ranged between 1 and 27. The optimal *P*-value was 0.0190 when the cut-off value was set at 3. **(B)** The correlation between sbTMB and OS in the POPLAR cohort. Patients with a low sbTMB or high sbTMB (sbTMB > 12) had a greater OS benefit after atezolizumab therapy than those with a moderate sbTMB. **(C)** The

alteration in each *P*-value when the cut-off value was changed in the OAK cohort. The cut-off value ranged between 1 and 27. The optimal *P*-value was 0.0003 when the cut-off value was set at 4. **(D)** The correlation between sbTMB and overall survival (OS) in the OAK cohort. Patients with a low sbTMB or high sbTMB (sbTMB > 17) had a greater OS benefit after atezolizumab therapy than those with a moderate sbTMB. **(E)** Absolute OS analysis between patients with bTMB ≤4 or >17 (*n*= 147) and those with 4< bTMB ≤17 (*n*= 177) (OS: unpaired t test *P*= 0.0002). **(F)** ROC curve for the correlation of sbTMB with response to atezolizumab in the OAK cohort. The AUC of OS response prediction was 0.64 (95% CI 0.55 to 0.68, null hypothesis test *P*= 0.0004). **(G) Left:** The nomogram showed the correlation between sbTMB and OS in POPLAR cohort. **Right:** The nomogram showed the correlation between sbTMB and OS in OAK cohort.

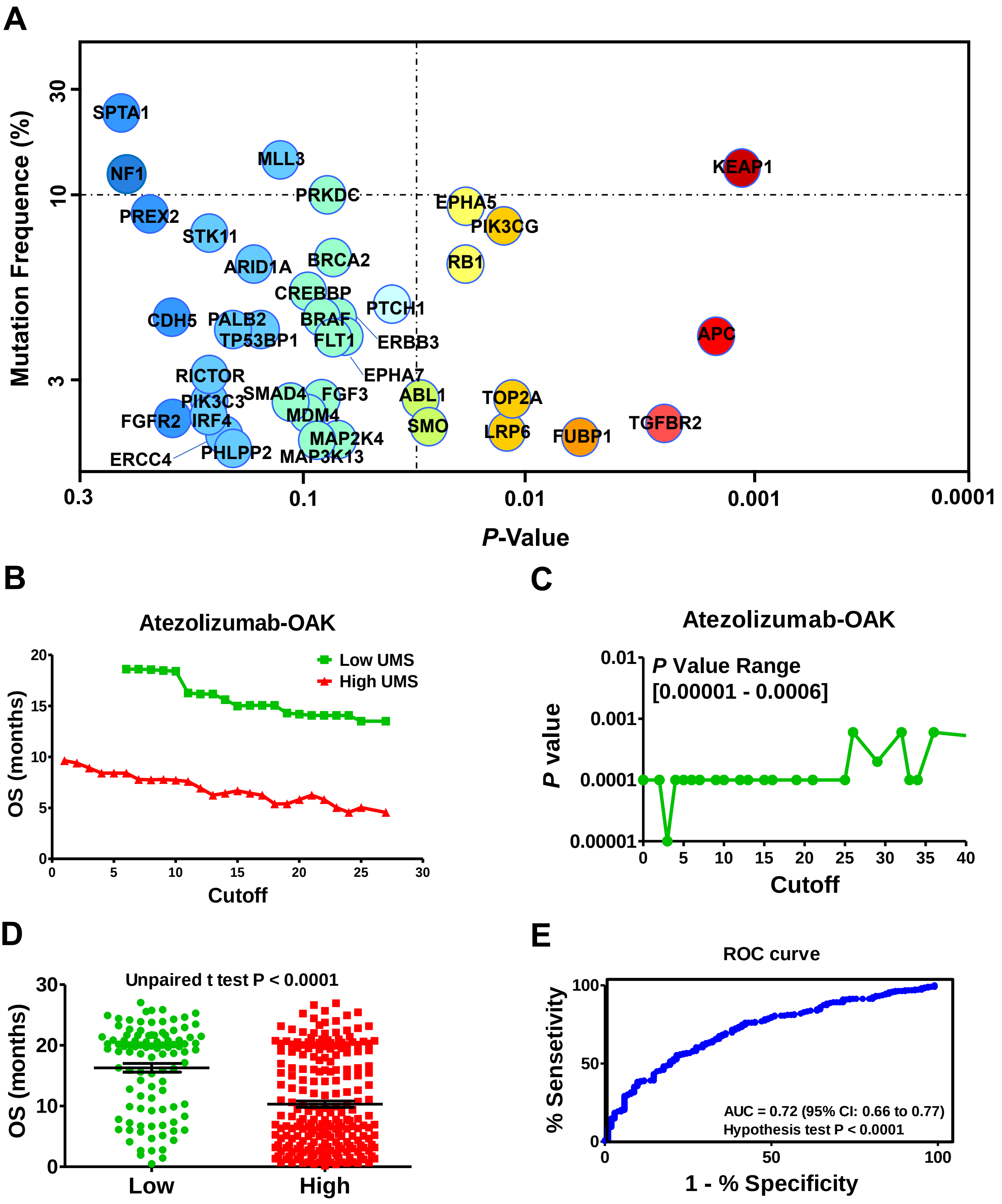
A
Mutation Frequence (%)
P-Value
SPTA1
NF1
MLL3
PRKDC
PREX2
STK11
ARID1A
BRCA2
CREBBP
PTCH1
CDH5
PALB2
BRAF
TP53BP1
FLT1
ERBB3
EPHA7
RICTOR
PIK3C3
SMAD4
FGF3
ABL1
MDM4
FGFR2
IRF4
SMO
MAP2K4
PHLPP2
MAP3K13
ERCC4
EPHA5
PIK3CG
RB1
TOP2A
LRP6
FUBP1
TGFBR2
KEAP1
APC
B
Atezolizumab-OAK
Low UMS
High UMS
OS (months)
Cutoff
C
Atezolizumab-OAK
P Value Range
[0.00001 - 0.0006]
P value
Cutoff
D
Unpaired t test P < 0.0001
OS (months)
Low
High
E
ROC curve
% Sensetivity
1 - % Specificity
AUC = 0.72 (95% CI: 0.66 to 0.77)
Hypothesis test P < 0.0001

Supplementary Figure 10. Cut-off analysis of the biomarker UMS for NSCLC patients in the OAK cohort who received atezolizumab. (A) The correlation analysis between mutated genes and OS. Mutated frequency and significant *P*-value were showed. **(B)** The correlation between the UMS and OS. Patients with a low UMS had a greater OS benefit after atezolizumab therapy than those with a high UMS. **(C)** The alteration in each *P*-value when the cut-off value was changed in the OAK cohort. The cut-off value ranged between 1 and 27. The optimal *P*-value was less than 0.00001 when the cut-off value was set at 3. **(D)** Absolute OS analysis between patients in the OAK cohort with a low UMS (n= 102) and a high UMS (n= 222) (OS: unpaired t test $P < 0.0001$). **(E)** ROC curve for the correlation of the UMS with response to docetaxel. The AUC of OS response prediction was 0.72 (95% CI 0.66 to 0.77, null hypothesis test $P < 0.0001$).

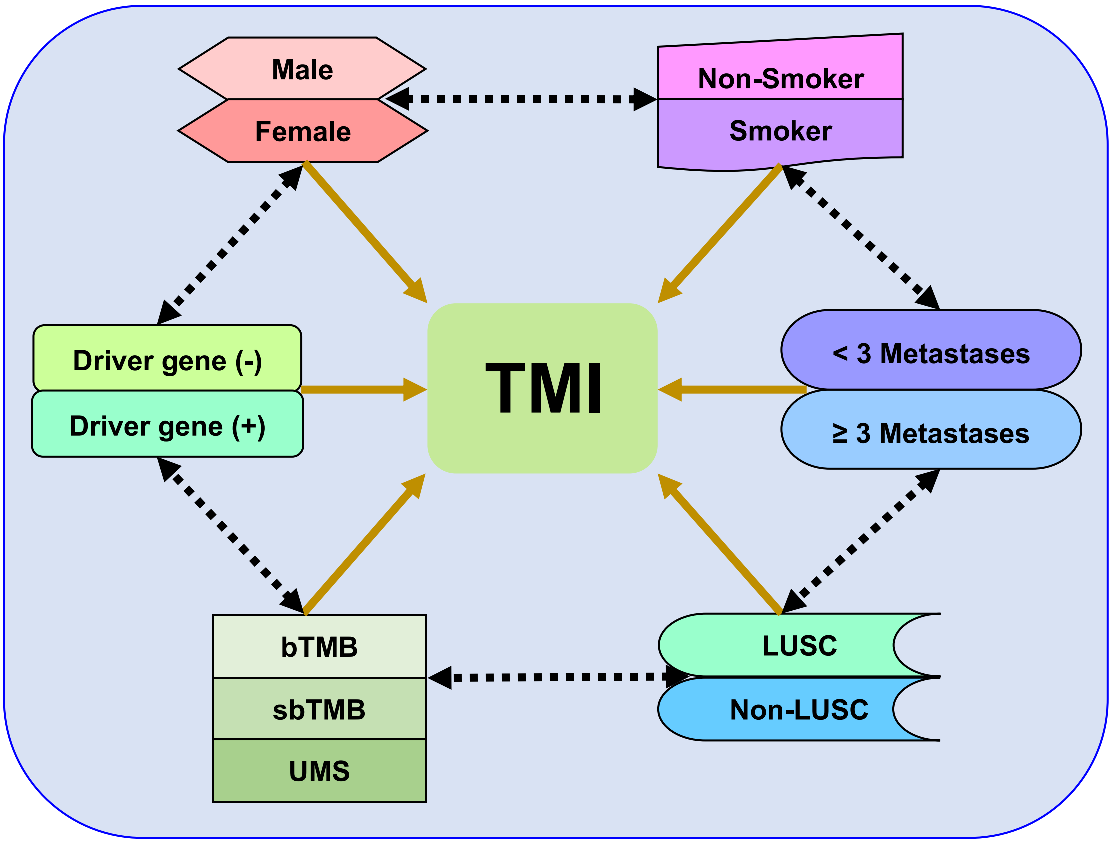

Supplementary Figure 11. Schematic diagram for generating the tumour mutation index. The tumour mutation index (TMI) is a comprehensive index that derives from three biomarkers: bTMB, sbTMB and UMS. Under each biomarker, the hazard ratio (HR) of each subgroup (male, female, smoker, non-smoker, LUSC, non-LUSC, driver gene (-), driver gene (+), <3 metastases, and ≥ 3 metastases) was calculated based on their stratification. According to the HRs of each subgroup under the biomarkers bTMB, sbTMB, and UMS, the comprehensive index defined as the TMI was calculated.

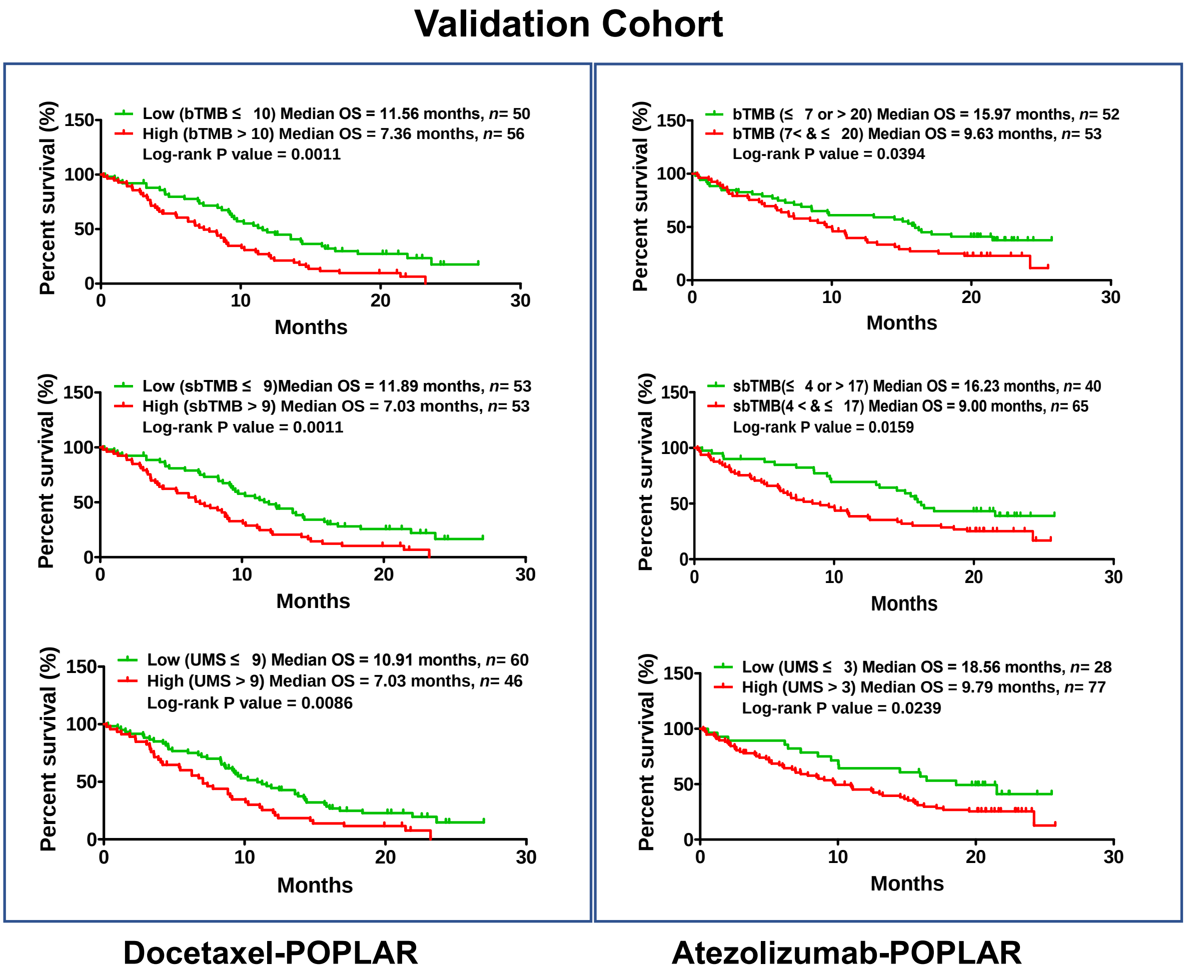

Supplementary Figure 12. Validation of the biomarkers (bTMB, sbTMB, UMS) validity in POPLAR cohort. Left: Kaplan-Meier plots of OS in NSCLC patients receiving docetaxel when using the biomarkers bTMB, sbTMB and UMS. The cut-off values used here is consistent with discovery cohort (OAK cohort). OS curves of responders and non-responders are shown on the top left, middle left, and bottom left. **Right:** Kaplan-Meier plots of OS in NSCLC patients receiving atezolizumab when using the biomarkers bTMB, sbTMB and UMS. The cut-off values used here is consistent with discovery cohort (OAK cohort). OS curves of responders and non-responders are shown on the top right, middle right, and bottom right.

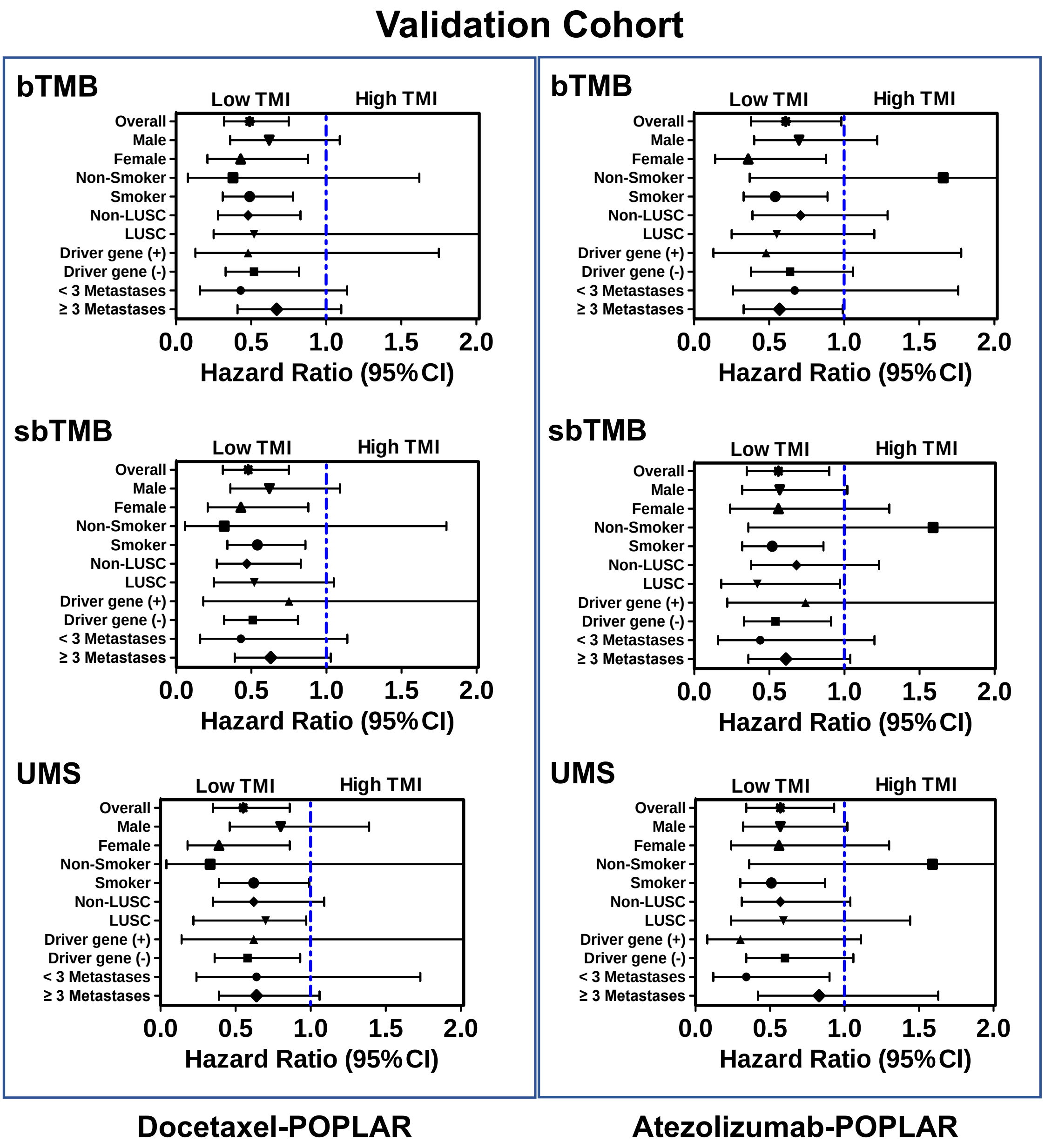

Supplementary Figure 13. Validation of the HRs via the biomarkers (bTMB, sbTMB, UMS)-based stratification in POPLAR cohort. Left: All patients received monotherapy of docetaxel in POPLAR cohort (validation cohort). The HRs of all patients and the corresponding subgroups via the biomarkers (bTMB, sbTMB, UMS)-based stratification. **Right:** All patients received monotherapy of atezolizumab in POPLAR cohort (validation cohort). The HRs of all patients and the corresponding subgroups via the biomarkers (bTMB, sbTMB, UMS)-based stratification.

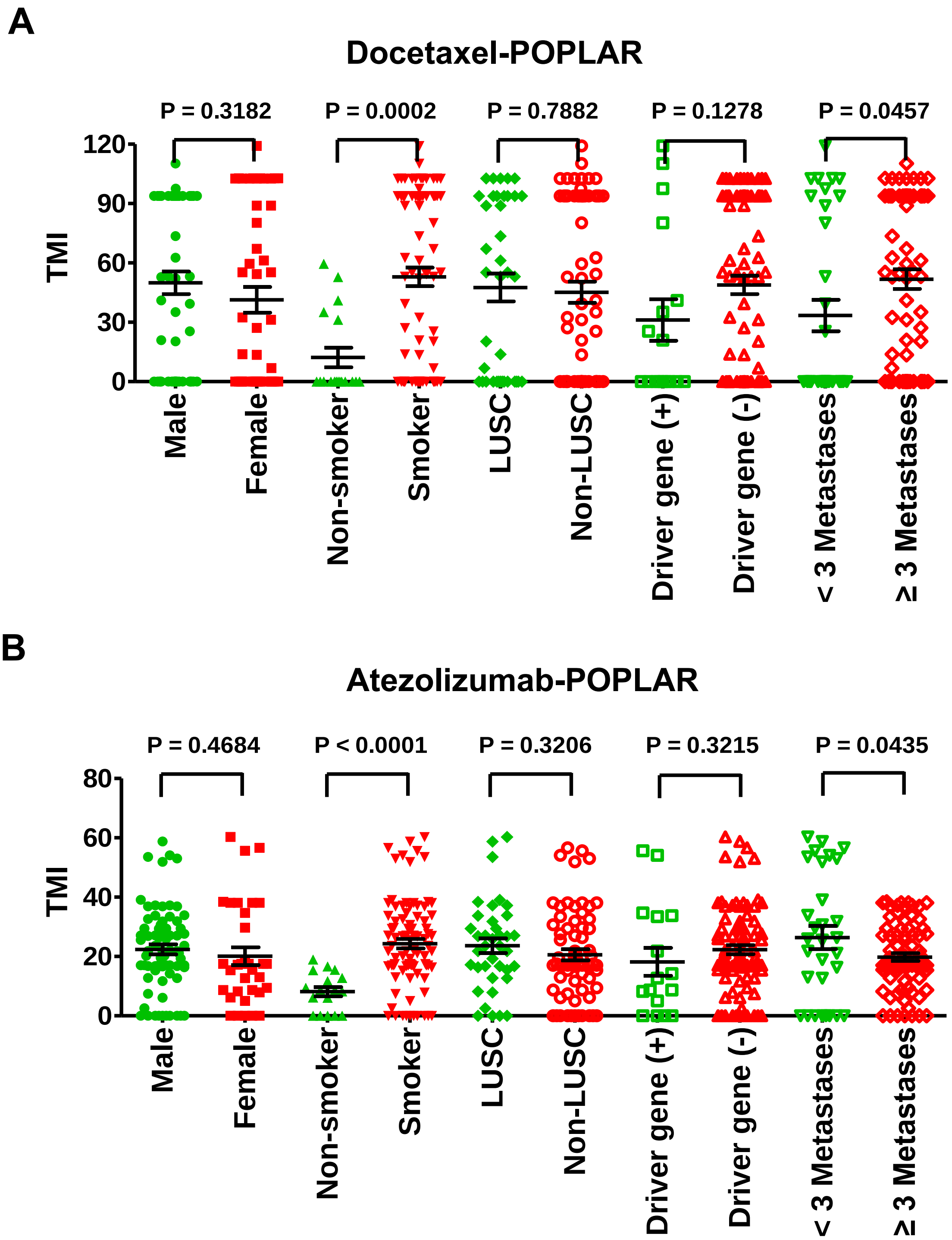

Supplementary Figure 14. Subgroup characteristics of the tumour mutation index in the validation cohort (POPLAR cohort). (A) Comparison of the differences between clinical characteristics. For patients who received docetaxel, there was no significant difference in three clinical characteristics (male vs female (*P*= 0.3108),

LUSC vs non-LUSC (*P*= 0.7882), and driver gene (-) vs driver gene (+) (*P*= 0.1278)); there was a remarkable difference in two clinical characteristics (smoker vs non-smoker (*P*= 0.0002), <3 metastases vs ≥ 3 metastases (*P*= 0.0457)). **(B)** For patients who received atezolizumab, there was no significant difference in three clinical characteristics (male vs female (*P*= 0.4684), LUSC vs non-LUSC (*P*= 0.3206), and driver gene (-) vs driver gene (+) (*P*= 0.3215)); there was a remarkable difference in two clinical characteristics (smoker vs non-smoker (*P* < 0.0001), <3 metastases vs ≥ 3 metastases (*P*= 0.0435)).

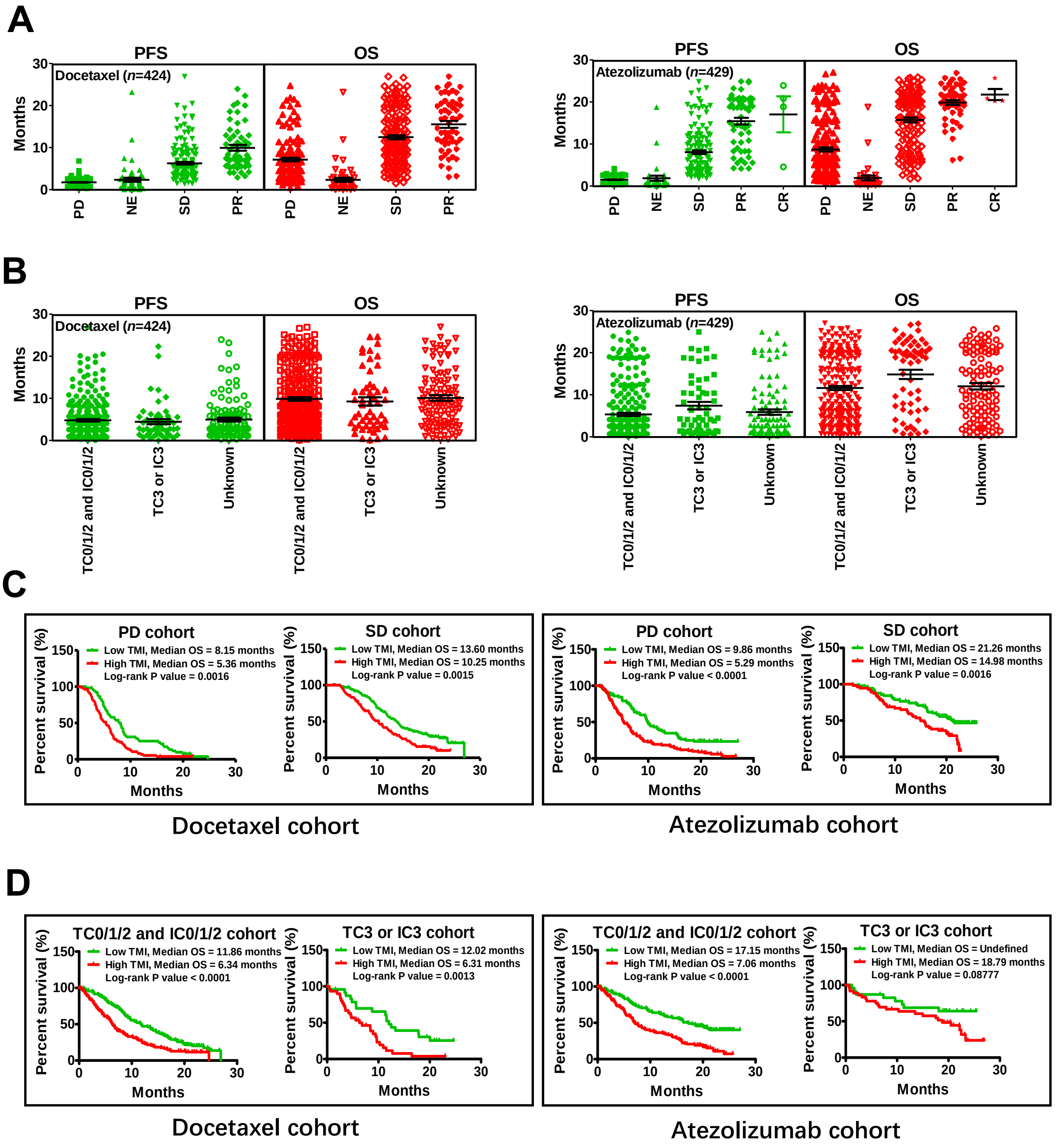

Supplementary Figure 15. Tumour mutation index stratification based on efficacy evaluation-based subgroups and PD-L1 expression-based subgroups. (A) Efficacy analysis of NSCLC patients who received monotherapy with docetaxel or atezolizumab. Patients who achieved PR or complete response (CR) received a greater OS benefit from therapy, while patients who were not evaluable for response (NE) received a lesser OS benefit from therapy. Some patients who achieved PD or SD received a good OS

benefit, while others did not. **(B)** Efficacy differences in patients with different PD-L1 expression levels after receiving monotherapy with docetaxel or atezolizumab. Patients classified as TC0/1/2 and IC0/1/2 or TC3 and IC3 or unknown received similar PFS and OS benefits from docetaxel therapy, while those classified as TC3 or IC3 received more PFS and OS benefits from atezolizumab therapy. **(C)** The TMI was used for to stratify efficacy (classified as PD or SD). Patients received monotherapy with docetaxel or atezolizumab. The TMI could be used to determine whether patients who achieved PD or SD would receive an OS benefit. **(D)** The TMI was used responders based on PD-L1 expression (defined as TC0/1/2 and IC0/1/2 or TC3 and IC3). Patients received monotherapy with docetaxel or atezolizumab. The TMI could be used to screen patients who would receive an OS benefit regardless of the PD-L1 expression status.

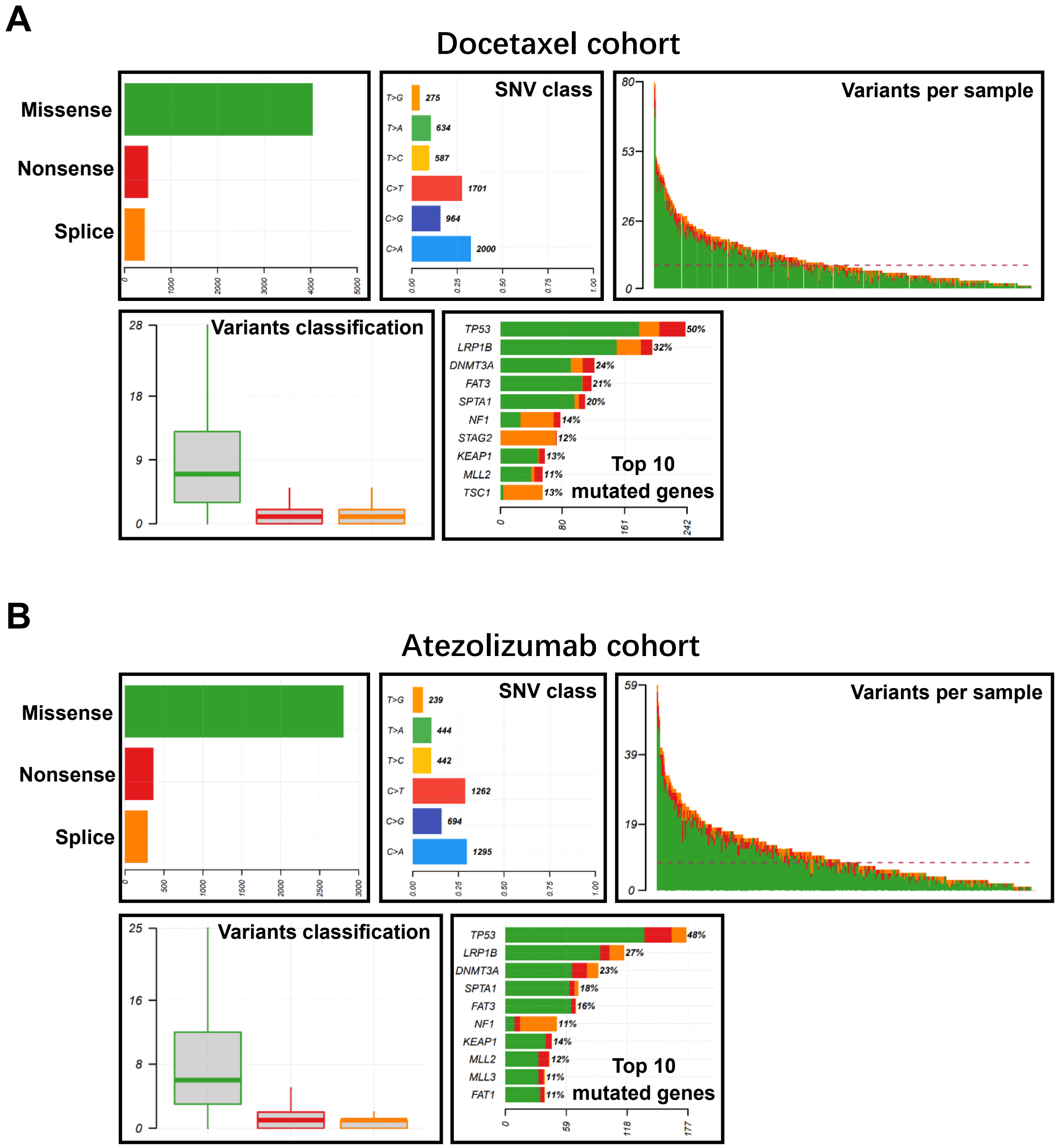

Supplementary Figure 16. Mutational profiling of patients who received docetaxel or atezolizumab. (A) In total, 424 patients were enrolled in the docetaxel cohort. **Top left:** Numbers of all types of mutations (missense, nonsense, and splice). **Top middle:** SNV class (C >A, C >G, C >T, T >C, T >A, and T >G). **Top right:** Number of variants

per sample. **Bottom left:** Variant classification. **Bottom right:** Top 10 mutated genes. **(B)** In total, 429 patients were enrolled in the atezolizumab cohort. **Top left:** Numbers of all types of mutations (missense, nonsense, and splice). **Top middle:** SNV class (C >A, C >G, C >T, T >C, T >A, and T >G). **Top right:** Variants per sample. **Bottom left:** Variant classification. **Bottom right:** Top 10 mutated genes.

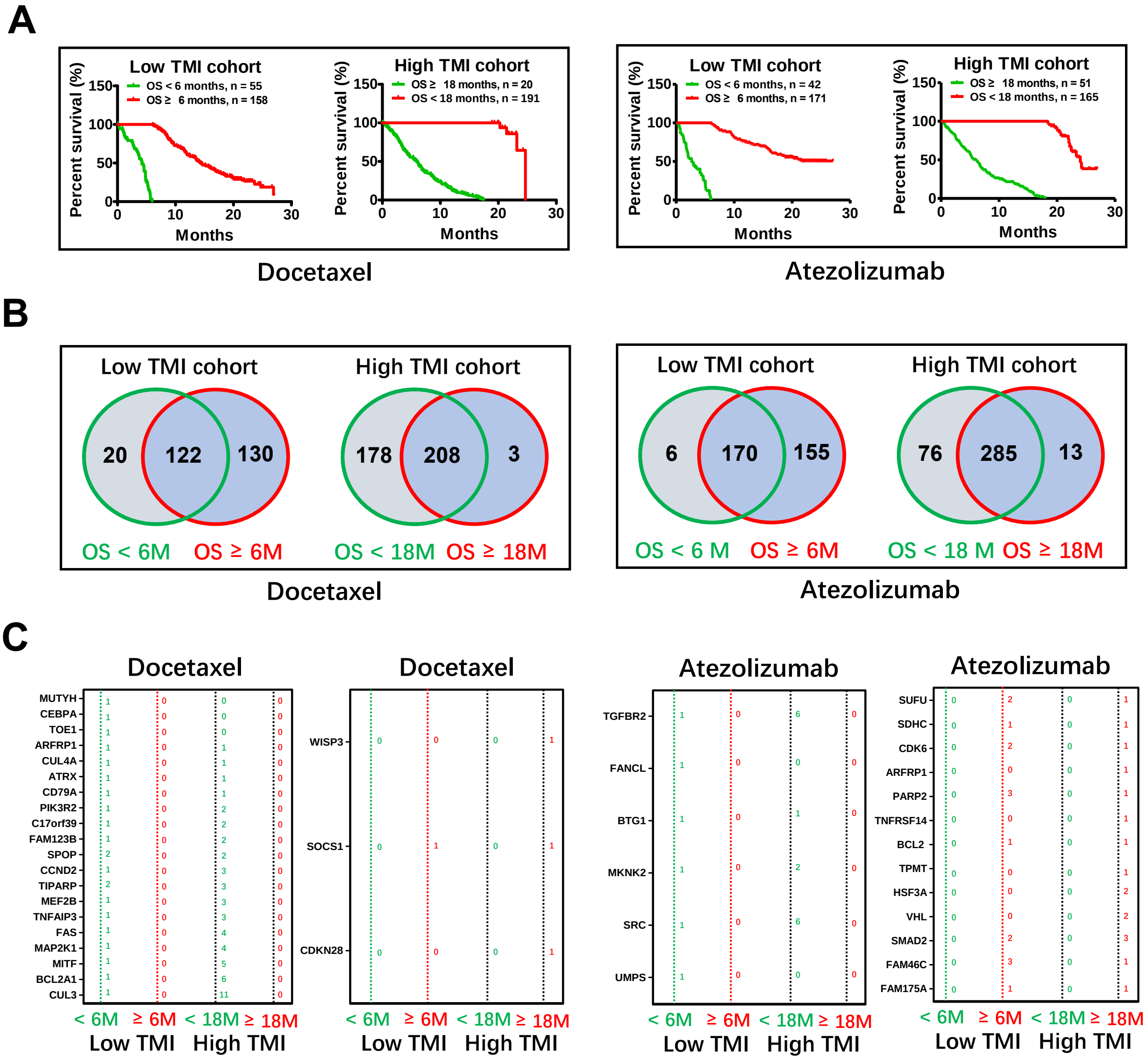

Supplementary Figure 17. Mutation differences under the tumour mutation index stratification system. (A) Kaplan-Meier plots of OS in NSCLC patients receiving docetaxel or atezolizumab. There were 55 docetaxel-treated patients with an OS duration < 6 months in the low TMI cohort and 20 docetaxel-treated patients with an OS duration ≥ 18 months in the high TMI cohort. There were 42 atezolizumab-treated patients with an OS duration < 6 months in the low TMI cohort and 51 atezolizumab-treated patients with an OS duration ≥ 18 months in the high TMI cohort. **(B)** Venn diagram of mutation differences. Twenty unique mutations existed in docetaxel-treated

patients with an OS duration < 6 months in the low TMI cohort, and 3 unique mutations existed in docetaxel-treated patients with an OS duration ≥ 18 months in the high TMI cohort. Six unique mutations existed in atezolizumab-treated patients with an OS duration < 6 months in the low TMI cohort, and 13 unique mutations existed in atezolizumab-treated patients with an OS duration ≥ 18 months in the high TMI cohort. **(C)** Mutated genes in each cohort.

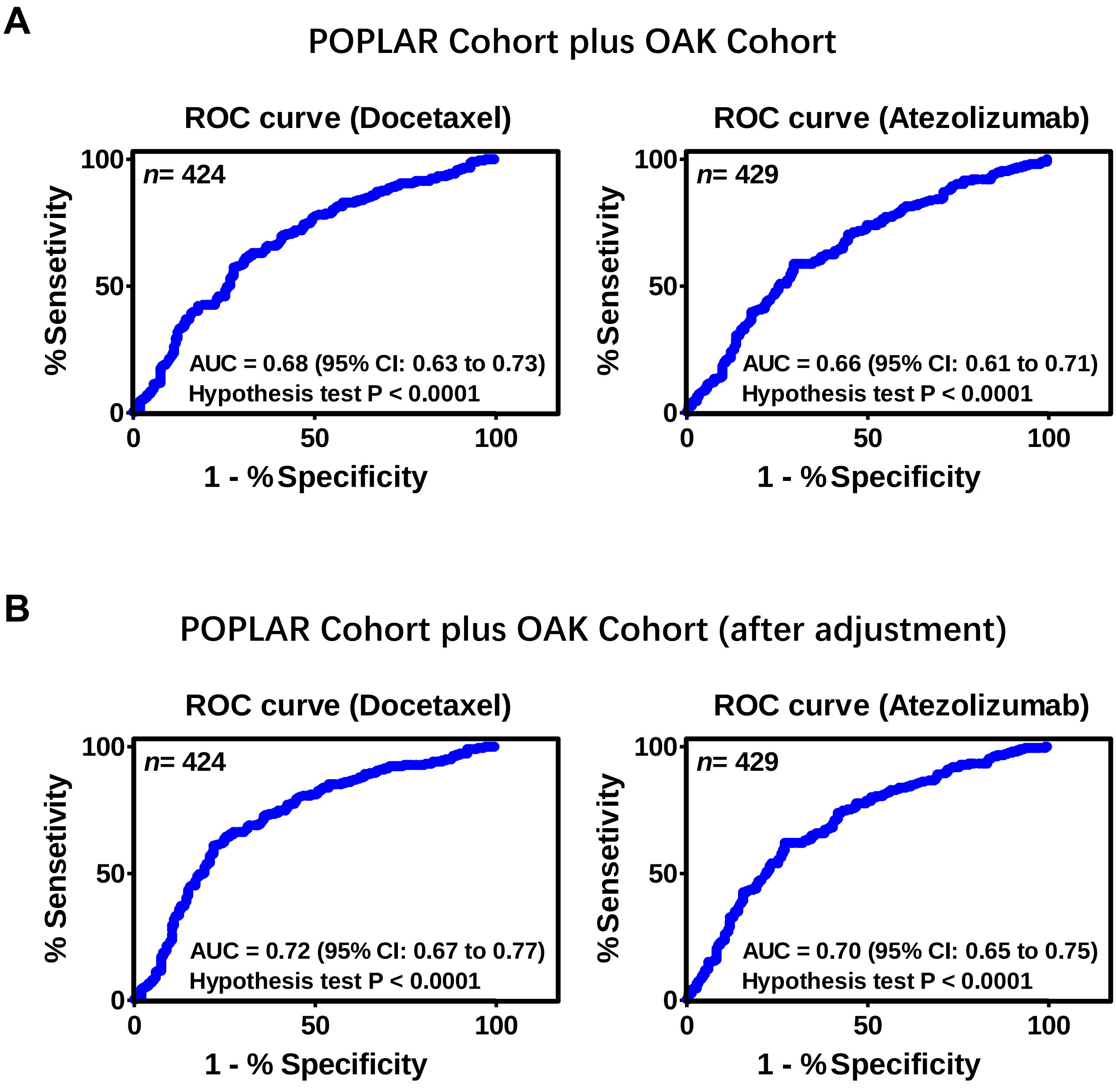

Supplementary Figure 18. ROC curve analysis used by TMI model to predict efficacy. (A) ROC curve analysis for the correlation of TMI and the prognosis of the therapies of docetaxel or atezolizumab. The AUC of OS outcome was 0.68 (95% CI 0.63 to 0.73, null hypothesis test *P*< 0.0001) in docetaxel cohort, and the AUC of OS outcome was 0.66 (95% CI 0.61 to 0.71, null hypothesis test *P*< 0.0001) in atezolizumab cohort. **(B)** ROC curve analysis for the correlation of TMI (after adjustment) and the prognosis of the therapies of docetaxel or atezolizumab. The AUC

of OS outcome was 0.72 (95% CI 0.67 to 0.77, null hypothesis test *P*< 0.0001) in docetaxel cohort, and the AUC of OS outcome was 0.70 (95% CI 0.65 to 0.75, null hypothesis test *P*< 0.0001) in atezolizumab cohort.

Supplementary Table 1. Subgroups responsive analysis using the biomarkers of bTMB, sbTMB, UMS in docetaxel-treated patients from OAK cohort (discovery cohort).

		**Median PFS (months)**			**ROC curve**		**Median OS (months)**			**ROC curve**	
		**bTMB (≤ 10)**	**bTMB (10 >)**	***P* value**	**AUC**	***P* value**	**bTMB (≤ 10)**	**bTMB (10 >)**	***P* value**	**AUC**	***P* value**
**bTMB**	**Male**	5.15	2.79	<0.0001	0.6865	<0.0001	11.53	6.57	0.0059	0.6554	0.0002
	**Female**	4.04	2.09	0.2309	0.5702	0.1782	11.70	6.80	0.0065	0.6283	0.0164
	**Non-Smoker**	4.17	2.15	0.0173	0.6858	0.0878	13.47	4.73	0.0906	0.6554	0.1721
	**Smoker**	4.50	2.79	<0.0001	0.6343	0.0001	11.33	6.57	0.0014	0.6353	0.0001
	**Non-LUSC**	4.76	2.86	0.0014	0.6185	0.0022	11.86	6.80	0.0024	0.6259	0.0012
	**LUSC**	4.14	1.91	0.0055	0.6531	0.0121	9.63	6.24	0.0338	0.6651	0.0068
	**Driver gene (+)**	4.50	2.86	0.0559	0.6427	0.0993	13.67	4.73	0.0562	0.6272	0.1420
	**Driver gene (-)**	4.30	2.79	0.0001	0.6313	0.0002	10.97	6.57	0.0011	0.6404	<0.0001
	**< 3 Metastases**	4.83	2.79	0.0128	0.6453	0.0070	15.64	6.80	<0.0001	0.7057	0.0001
	**≥ 3 Metastases**	4.21	2.79	0.0006	0.6236	0.0027	9.82	6.44	0.0995	0.5957	0.0202
**sbTMB**		**sbTMB (≤ 9)**	**sbTMB (> 9)**	***P* value**	**AUC**	***P* value**	**sbTMB (≤ 9)**	**sbTMB (> 9)**	***P* value**	**AUC**	***P* value**
	**Male**	4.80	2.79	<0.0001	0.6808	<0.0001	11.53	6.44	0.0011	0.6646	<0.0001
	**Female**	4.04	2.79	0.4413	0.5581	0.2878	11.70	6.80	0.0046	0.6258	0.0215
	**Non-Smoker**	4.17	2.15	0.0189	0.7256	0.1401	13.60	6.45	0.0011	0.7439	0.1107
	**Smoker**	4.34	2.79	0.0003	0.6300	0.0002	10.87	6.57	0.0004	0.6404	<0.0001
	**Non-LUSC**	4.70	2.86	0.0021	0.6268	0.0012	12.02	6.80	0.0005	0.6325	0.0007
	**LUSC**	4.14	1.94	0.0174	0.6220	0.0443	8.61	6.24	0.0080	0.6735	0.0042
	**Driver gene (+)**	4.34	2.18	0.1152	0.6448	0.1135	15.24	4.53	0.0020	0.7239	0.0143
	**Driver gene (-)**	4.17	2.79	0.0005	0.6244	0.0005	9.82	6.80	0.0008	0.6307	0.0002
	**< 3 Metastases**	4.73	2.86	0.0562	0.6168	0.0335	15.24	6.80	<0.0001	0.7198	<0.0001
	**≥ 3 Metastases**	4.17	2.69	0.0007	0.6342	0.0011	9.76	6.57	0.0612	0.5988	0.0165
**UMS**		**UMS (≤ 9)**	**UMS (> 9)**	***P* value**	**AUC**	***P* value**	**UMS (≤ 9)**	**UMS (> 9)**	***P* value**	**AUC**	***P* value**
	**Male**	5.55	2.96	0.0001	0.6690	0.0001	13.01	6.87	<0.0001	0.6804	<0.0001
	**Female**	4.40	2.79	0.0113	0.6357	0.0093	14.03	6.80	0.0006	0.6817	0.0005
	**Non-Smoker**	4.17	2.81	0.0665	0.6351	0.1689	13.47	5.65	0.0976	0.6604	0.1032
	**Smoker**	5.32	2.89	<0.0001	0.6607	<0.0001	14.09	6.80	<0.0001	0.6818	<0.0001
	**Non-LUSC**	5.13	2.89	0.0002	0.6400	0.0003	14.09	6.80	<0.0001	0.6804	<0.0001
	**LUSC**	4.30	2.83	0.0221	0.6624	0.0146	8.80	7.26	0.0054	0.6407	0.0342
	**Driver gene (+)**	4.50	2.69	0.0445	0.6960	0.0181	16.26	4.70	0.0005	0.7786	0.0008
	**Driver gene (-)**	5.32	2.89	<0.0001	0.6363	0.0002	11.86	6.87	<0.0001	0.6545	<0.0001
	**< 3 Metastases**	5.68	2.83	0.0004	0.6926	0.0003	16.62	6.87	<0.0001	0.7089	<0.0001
	**≥ 3 Metastases**	4.21	2.89	0.0044	0.6120	0.0112	11.33	6.80	0.0113	0.6449	0.0010

bTMB: Blood tumor mutation burden; sbTMB: sensitive blood tumor mutation burden; UMS: Unfavorable mutation score; LUSC: lung squamous carcinoma.

Supplementary Table 2. Subgroups responsive analysis using the biomarkers of bTMB, sbTMB, UMS in atezolizumab-treated patients from OAK cohort (discovery cohort).

		Median PFS (months)			ROC curve		Median OS (months)			ROC curve	
		bTMB (≤ 7 & > 20)	bTMB (7> & ≤ 20)	*P* value	AUC	*P* value	bTMB (≤ 7 & > 20)	bTMB (7> & ≤ 20)	*P* value	AUC	*P* value
**bTMB**	**Male**	2.96	1.41	0.0006	0.6454	0.0004	15.67	7.33	< 0.0001	0.6385	0.0007
	**Female**	2.94	2.89	0.7314	0.5464	0.4208	16.26	8.90	0.0109	0.5842	0.1444
	**Non-Smoker**	2.79	2.43	0.1558	0.6078	0.1820	17.15	9.593	0.1997	0.5289	0.7207
	**Smoker**	4.008	1.544	0.0011	0.6323	0.0003	15.90	7.326	<0.0001	0.6455	<0.0001
	**Non-LUSC**	3.943	1.544	0.0139	0.6296	0.0010	16.99	9.166	0.0012	0.6307	0.0009
	**LUSC**	2.858	1.577	0.0133	0.6275	0.0409	12.32	7.228	0.1195	0.6082	0.0829
	**Driver gene (+)**	4.041	2.382	0.4917	0.5615	0.3277	11.30	16.03	0.8120	0.5235	0.7750
	**Driver gene (-)**	3.91	1.511	0.0005	0.6418	<0.0001	16.3	7.326	<0.0001	0.6447	<0.0001
	**< 3 Metastases**	4.205	1.610	0.0188	0.6400	0.0069	20.24	8.444	0.0011	0.6435	0.0056
	**≥ 3 Metastases**	2.793	1.544	0.0357	0.6203	0.0048	13.50	7.326	0.0132	0.6185	0.0054
**sbTMB**		sbTMB (≤ 4 & > 17)	sbTMB (≤ 4 & > 17)	*P* value	AUC	*P* value	sbTMB (≤ 4 & > 17)	sbTMB (≤ 4 & > 17)	*P* value	AUC	*P* value
	**Male**	4.24	1.51	<0.0001	0.6713	<0.0001	15.93	8.28	0.0134	0.6165	0.0048
	**Female**	4.07	2.69	0.1320	0.6160	0.0284	18.60	8.90	0.0036	0.6017	0.0532
	**Non-Smoker**	4.04	1.61	0.0206	0.7228	0.0682	18.69	9.59	0.0690	0.5808	0.2847
	**Smoker**	4.19	1.61	<0.0001	0.6413	0.0001	16.26	7.89	0.0009	0.6257	0.0005
	**Non-LUSC**	4.107	1.577	<0.0001	0.6724	<0.0001	18.69	10.61	0.0009	0.6179	0.0017
	**LUSC**	2.858	1.61	0.0133	0.6390	0.0294	13.47	7.08	0.0868	0.6224	0.0551
	**Driver gene (+)**	4.107	2.333	0.0791	0.6693	0.04	13.03	11.03	0.6539	0.5462	0.5748
	**Driver gene (-)**	4.172	1.511	<0.0001	0.6650	<0.0001	17.15	7.885	0.0001	0.6275	0.0003
	**< 3 Metastases**	5.667	2.004	0.0004	0.6731	0.0005	Undefined	9.429	0.0092	0.6095	0.0289
	**≥ 3 Metastases**	2.727	1.61	0.0709	0.5985	0.0609	15.90	7.326	0.0082	0.6195	0.0047
**UMS**		UMS (≤ 3)	UMS (> 3)	*P* value	AUC	*P* value	UMS (≤ 3)	UMS (> 3)	*P* value	AUC	*P* value
	**Male**	2.76	1.676	0.0721	0.5769	0.1102	Undefined	8.411	<0.0001	0.7297	<0.0001
	**Female**	4.205	2.776	0.0803	0.6262	0.0171	Undefined	8.903	<0.0001	0.6944	0.0002
	**Non-Smoker**	2.76	2.563	0.5526	0.5434	0.6889	18.69	8.903	0.0449	0.6029	0.1772
	**Smoker**	4.567	1.84	0.0011	0.6508	0.0002	Undefined	8.411	<0.0001	0.7577	<0.0001
	**Non-LUSC**	4.008	2.251	0.1142	0.5818	0.0351	Undefined	9.462	<0.0001	0.6888	<0.0001
	**LUSC**	12.16	2.004	0.0017	0.7651	0.0024	Undefined	7.589	0.0004	0.8051	0.0005
	**Driver gene (+)**	4.041	2.694	0.9117	0.5379	0.6574	14.92	6.801	0.3691	0.6751	0.0404
	**Driver gene (-)**	4.172	1.708	0.0013	0.6327	0.0005	Undefined	8.444	<0.0001	0.7213	<0.0001
	**< 3 Metastases**	4.485	2.727	0.0365	0.6404	0.0063	Undefined	8.444	<0.0001	0.7288	<0.0001
	**≥ 3 Metastases**	2.776	1.725	0.188	0.5806	0.0911	Undefined	8.411	<0.0001	0.6823	0.0002

bTMB: Blood tumor mutation burden; sbTMB: sensitive blood tumor mutation burden; UMS: Unfavorable mutation score; LUSC: lung squamous carcinoma.

Supplementary Table 3. Subgroups responsive analysis using the biomarkers of bTMB, sbTMB, UMS, and TMI in docetaxel-treated patients from POPLAR cohort (validation cohort).

		Median PFS (months)			Median OS (months)		
**bTMB**		**bTMB (≤ 10)**	**bTMB (10 >)**	***P* value**	**bTMB (≤ 10)**	**bTMB (10 >)**	***P* value**
	**Male**	4.01	2.86	0.2310	9.36	8.31	0.0951
	**Female**	5.55	1.58	0.0454	13.57	5.42	0.0219
	**Non-Smoker**	5.59	3.35	0.0201	13.57	8.28	0.1853
	**Smoker**	2.92	1.87	0.1214	9.72	7.03	0.0092
	**Non-LUSC**	5.57	2.71	0.0044	11.56	7.56	0.0088
	**LUSC**	2.83	2.17	0.7482	10.1	6.87	0.0709
	**Driver gene (+)**	7.75	4.07	0.0004	12.4	10.6	0.3204
	**Driver gene (-)**	3.52	2.27	0.1870	11.24	6.72	0.0060
	**< 3 Metastases**	5.85	3.88	0.2126	15.92	8.57	0.0927
	**≥ 3 Metastases**	3.42	1.87	0.1170	8.89	6.74	0.1128
**sbTMB**		**sbTMB (≤ 9)**	**sbTMB (> 9)**	***P* value**	**sbTMB (≤ 9)**	**sbTMB (> 9)**	***P* value**
	**Male**	4.01	2.86	0.3400	9.36	7.75	0.0932
	**Female**	4.88	1.49	0.0523	13.68	4.94	0.0217
	**Non-Smoker**	5.57	3.01	0.0468	12.98	7.26	0.1950
	**Smoker**	2.92	1.87	0.1763	9.72	6.74	0.0096
	**Non-LUSC**	5.55	2.66	0.0133	11.89	6.74	0.0086
	**LUSC**	2.83	2.17	0.7482	10.10	6.87	0.0709
	**Driver gene (+)**	6.64	4.16	0.0215	11.81	11.5	0.7448
	**Driver gene (-)**	3.94	1.87	0.1892	11.56	6.70	0.0047
	**< 3 Metastases**	5.85	3.88	0.2126	15.92	8.57	0.0927
	**≥ 3 Metastases**	3.94	1.72	0.1715	9.13	6.47	0.0640
**UMS**		**UMS (≤ 9)**	**UMS (> 9)**	***P* value**	**UMS (≤ 9)**	**UMS (> 9)**	***P* value**
	**Male**	4.01	2.86	0.3400	9.36	7.75	0.0932
	**Female**	4.88	1.49	0.0523	13.68	4.94	0.0217
	**Non-Smoker**	5.57	3.01	0.0468	12.98	7.26	0.1950
	**Smoker**	2.92	1.87	0.1763	9.72	6.74	0.0096
	**Non-LUSC**	5.55	2.66	0.0133	11.89	6.74	0.0086
	**LUSC**	2.83	2.17	0.7482	10.1	6.87	0.0709
	**Driver gene (+)**	6.64	4.16	0.0215	11.81	11.5	0.7448
	**Driver gene (-)**	3.94	1.87	0.1892	11.56	6.70	0.0047
	**< 3 Metastases**	5.85	3.88	0.2126	15.92	8.57	0.0927
	**≥ 3 Metastases**	3.94	1.72	0.1715	9.13	6.47	0.0640
**TMI**		**Low TMI**	**High TMI**	***P* value**	**Low TMI**	**High TMI**	***P* value**
	**Male**	4.01	2.86	0.3600	9.36	7.03	0.0766
	**Female**	5.55	1.58	0.0867	13.8	4.40	0.0168
	**Non-Smoker**	5.57	2.35	0.0231	12.98	5.36	0.1709
	**Smoker**	2.92	2.22	0.2301	10.32	6.47	0.0076
	**Non-LUSC**	5.55	1.87	0.0388	11.89	6.24	0.0139
	**LUSC**	2.83	2.56	0.6215	11.56	6.70	0.0375
	**Driver gene (+)**	6.64	3.50	0.0309	12.98	6.18	0.6985
	**Driver gene (-)**	3.48	1.72	0.2201	11.24	6.24	0.0043
	**< 3 Metastases**	5.85	3.88	0.4528	15.92	7.75	0.1839
	**≥ 3 Metastases**	3.98	1.58	0.1361	9.18	6.24	0.0289

bTMB: Blood tumor mutation burden; sbTMB: sensitive blood tumor mutation burden; UMS: Unfavorable mutation score; TMI: Tumour mutation index; LUSC: lung squamous carcinoma.

Supplementary Table 4. Subgroups responsive analysis using the biomarkers of bTMB, sbTMB, UMS, and TMI in atezolizumab-treated patients from POPLAR cohort (validation cohort).

		Median PFS (months)			Median OS (months)		
**bTMB**		**bTMB (≤ 7 & > 20)**	**bTMB (7> & ≤ 20)**	***P* value**	**bTMB (≤ 7 & > 20)**	**bTMB (7> & ≤ 20)**	***P* value**
	**Male**	4.27	1.51	0.0643	10.02	10.05	0.4301
	**Female**	2.94	1.45	0.7163	16.1	8.44	0.1055
	**Non-Smoker**	2.69	4.24	0.3343	14.49	20.75	0.3750
	**Smoker**	4.37	1.48	0.0268	14.05	7.10	0.0472
	**Non-LUSC**	2.73	1.43	0.3156	15.47	10.25	0.2654
	**LUSC**	6.13	2.02	0.0889	10.28	7.85	0.3487
	**Driver gene (+)**	2.66	1.4	0.1066	16.43	8.26	0.0987
	**Driver gene (-)**	3.65	2.02	0.1482	13.75	8.97	0.2669
	**< 3 Metastases**	5.52	2.53	0.8503	18.56	11.01	0.6458
	**≥ 3 Metastases**	2.69	1.45	0.0493	11.01	7.26	0.1037
**sbTMB**		**sbTMB (≤ 4 & > 17)**	**sbTMB (≤ 4 & > 17)**	***P* value**	**sbTMB (≤ 4 & > 17)**	**sbTMB (≤ 4 & > 17)**	***P* value**
	**Male**	5.01	1.48	0.0421	13.86	7.56	0.1871
	**Female**	2.94	2.53	0.7519	16.1	8.44	0.1091
	**Non-Smoker**	2.69	4.17	0.5345	14.49	20.75	0.2193
	**Smoker**	5.75	1.51	0.0264	15.77	7.29	0.0131
	**Non-LUSC**	3.15	1.48	0.2383	15.47	9.51	0.3166
	**LUSC**	6.77	2.14	0.1194	15.87	7.26	0.0652
	**Driver gene (+)**	1.51	1.41	0.3800	16.43	10.78	0.2489
	**Driver gene (-)**	4.14	2.14	0.0915	15.47	7.29	0.0775
	**< 3 Metastases**	6.77	2.66	0.4740	21.13	10.48	0.0769
	**≥ 3 Metastases**	2.79	1.48	0.0789	13.24	7.10	0.1747
**UMS**		**UMS (≤ 3)**	**UMS (> 3)**	***P* value**	**UMS (≤ 3)**	**UMS (> 3)**	***P* value**
	**Male**	6.14	1.54	0.0780	18.07	8.20	0.0314
	**Female**	4.16	1.51	0.3186	17.26	13.01	0.4425
	**Non-Smoker**	3.15	1.46	0.1994	15.97	14.93	0.5435
	**Smoker**	6.77	1.58	0.0591	18.56	9.00	0.0141
	**Non-LUSC**	4.37	1.51	0.0593	19.14	9.79	0.0679
	**LUSC**	6.14	2.63	0.2890	12.96	7.87	0.2522
	**Driver gene (+)**	6.51	1.38	0.0049	20.55	9.00	0.0645
	**Driver gene (-)**	4.85	2.66	0.1243	15.92	9.66	0.0809
	**< 3 Metastases**	8.54	1.41	0.0193	20.14	9.00	0.0296
	**≥ 3 Metastases**	2.14	2.10	0.5698	10.05	9.66	0.5900
**TMI**		**Low TMI**	**High TMI**	***P* value**	**Low TMI**	**High TMI**	***P* value**
	**Male**	4.99	1.43	0.0183	12.78	7.10	0.0459
	**Female**	3.15	1.97	0.2445	16.23	7.41	0.0164
	**Non-Smoker**	2.69	NA	NA	15.97	NA	NA
	**Smoker**	5.63	1.43	0.0039	15.54	7.10	0.0163
	**Non-LUSC**	3.15	1.40	0.0322	15.97	7.10	0.0033
	**LUSC**	6.13	1.99	0.1091	10.5	7.10	0.3334
	**Driver gene (+)**	2.89	1.41	0.1768	18.07	9.00	0.0446
	**Driver gene (-)**	4.21	1.51	0.0168	15.47	6.93	0.0148
	**< 3 Metastases**	9.45	1.43	0.0147	20.17	8.79	0.0186
	**≥ 3 Metastases**	2.69	1.46	0.0907	11.07	6.51	0.0231

bTMB: Blood tumor mutation burden; sbTMB: sensitive blood tumor mutation burden; UMS: Unfavorable mutation score; TMI: Tumour mutation index; LUSC: lung squamous carcinoma; NA: no defined patient.
